# Trans-scale spin Seebeck effect in nanostructured bulk composites based on magnetic insulator

**DOI:** 10.1038/s41467-026-75232-0

**Published:** 2026-07-21

**Authors:** Sang J. Park, Keisuke Hirata, Hossein Sepehri-Amin, Fuyuki Ando, Takamasa Hirai, Ken-ichi Uchida

**Affiliations:** 1 National Institute for Materials Science, Tsukuba, Japan; 2 Toyota Technological Institute, Nagoya, Japan; 3 Department of Advanced Materials Science, Graduate School of Frontier Sciences, The University of Tokyo, Kashiwa, Japan

**Keywords:** Thermoelectrics, Magnetic properties and materials, Magnetic properties and materials

## Abstract

The spin Seebeck effect enables thermoelectric conversion through thermally generated spin currents in magnetic materials, offering a promising transverse geometry for scalable devices. However, conventional spin Seebeck devices are confined to nanoscale thin-film architectures, with significantly restricted output power due to the intrinsic constraints of spin and magnon diffusion lengths. Here, we demonstrate a trans-scale spin Seebeck effect using nanostructured bulk composites composed of Pt-coated yttrium iron garnet powders fabricated via dynamic powder sputtering and low-temperature sintering. The resulting three-dimensional composites exhibit continuous Pt channels and robust mechanical integrity. Transverse thermoelectric measurements reveal isotropic spin Seebeck signals at the bulk scale. Power analysis indicates that the three-dimensional architecture enables scalable volumetric thermoelectric power generation beyond diffusion-limited thin-film spin Seebeck geometries. This work establishes a scalable platform for spin Seebeck thermoelectric conversion, bridging nanoscale spin caloritronics with macroscopic device integration.

## Abstract (web summary)

This study demonstrates a three-dimensional bulk composite architecture based on magnetic insulators that extends spin Seebeck thermoelectric conversion beyond thin-film interfaces.

## Introduction

The spin Seebeck effect (SSE) enables the generation of electrical voltage via thermally driven spin currents (or magnons) in magnetically ordered materials^1–4^. In the SSE, a temperature gradient applied across a ferro(i)magnetic material (FM) excites spin currents, which are then pumped into an adjacent normal metal (NM). There, they are converted into a transverse electric field through the inverse spin Hall effect (ISHE)^5,6^. Owing to the orthogonal relationship between the heat flux and the induced electric field, the SSE is classified as a transverse thermoelectric effect^7–10^. Since its discovery in 2008^11^, the SSE has been a central topic of interest in both spintronics and thermoelectrics, serving as a platform for investigating fundamental transport physics^1–3,8–20^ and as a basis for thermoelectric energy harvesting applications^1–3,7–11,21–27^, based on thermally generated spin currents.

**Fig. 1:**
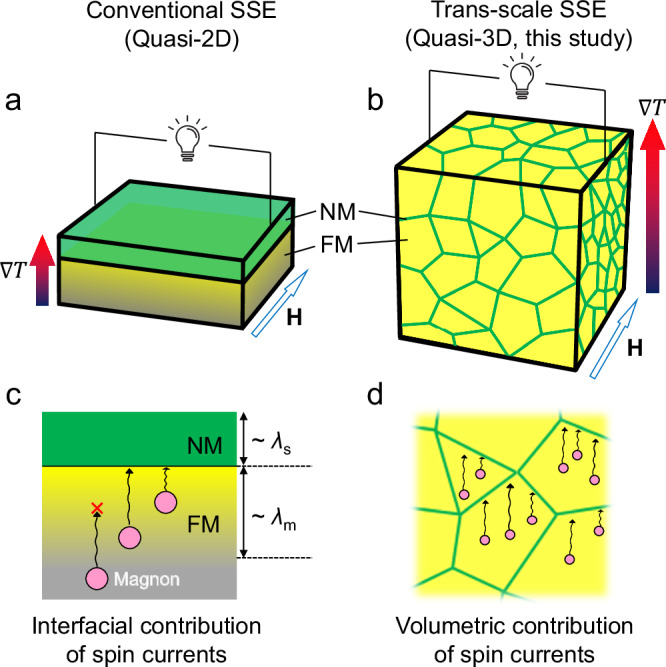
Conceptual comparison between conventional thin-film-based spin Seebeck effect (SSE) and the proposed trans-scale SSE. **a** Schematic of a typical quasi-two-dimensional SSE device composed of a ferro(i)magnetic material (FM) and a normal metal (NM) layer. The output electric field is generated orthogonal to the applied thermal gradient ($\nabla T$) and magnetic field (**H**). **b** Schematic of a trans-scale SSE device, realized via a bulk composite of NM-coated FM domains. **c** In the conventional SSE device, the device length scale is determined by spin diffusion length of NM *λ*_s_ and magnon diffusion length of FM *λ*_m_. Here, only spin currents and magnons that reach the FM/NM interface contribute to SSE, limiting device performance. **d** In the trans-scale SSE, distributed NM channels allow efficient local conversion of magnons throughout the FM volume, enhancing effective spin current utilization.

SSE-based thermoelectric energy harvesting has been proposed as a solution to the limitations of conventional thermoelectric devices, which rely on the longitudinal Seebeck effect to generate a charge current along the direction of a temperature gradient^28–31^. These longitudinal devices typically adopt π-shaped geometries with multiple n-type and p-type thermoelectric legs interconnected by electrodes such as solder. Such complex architectures hinder manufacturability, scalability, and overall device-level efficiency^7,32^. In contrast, SSE devices utilize simplified transverse configurations that eliminate the need for electrically and thermally sensitive junctions (Fig. 1a)^7–9^. This architectural simplicity enhances the mechanical robustness and scalability of devices, while also enabling more flexible material design. More importantly, the SSE enables thermoelectric conversion even in ferro(i)magnetic insulators (FMI), which are inaccessible to conventional thermoelectrics relying on mobile charge carriers. This unique capability allows SSE-based devices to harvest waste heat from insulating materials, thereby expanding the range of usable thermal sources beyond that of traditional approaches. In particular, the independent generation and propagation of spin and charge currents in the FM and NM layers, respectively, allow for separate optimization of each material, circumventing intrinsic trade-offs in material parameters, such as those dictated by the Wiedemann-Franz law^28^. These advantages make SSE-based devices promising candidates for scalable and reliable thermoelectric energy harvesting, despite their relatively low thermopower to date.

Over the past decades, numerous efforts have been made to enhance the SSE by improving energy conversion efficiencies within the FM and NM, and at their interface, for example, through magnon temperature manipulation^16^, the use of topological materials^33^, and interfacial engineering^22,34^. However, the practical implementation of SSE remains challenging, primarily due to the limited power output resulting from the hetero-structured nature of thin-film FM/NM devices (Fig. 1a). The device thickness is fundamentally constrained by the characteristic length of spin transport, namely the spin diffusion length (*λ*_s_), over which the spin angular momentum decays. As a result, the optimal thickness of the NM layer is typically limited to a few nanometers (e.g., less than 5 nm for sputtered Pt^35^), leading to high internal resistance and reduced output power.

The effective FM thickness is similarly governed by the magnon diffusion length (*λ*_m_) particularly in FMI, where spin angular momentum is carried solely by magnons, i.e., collective dynamics of localized magnetic moments^36–38^. For instance, yttrium iron garnet (YIG), a widely studied FMI known for its exceptionally low Gilbert damping constant (<10^−4^)^39^, exhibits *λ*_m_ of ~10 µm at room temperature^40,41^, which is significantly longer than that of ferro(i)magnetic metals (FMMs), e.g., 3.5 nm for sputtered Ni^42^. Nevertheless, these length scales remain microscopic, limiting device configurations to quasi-two-dimensional thin-film geometries (Fig. 1a), where only narrow volumes of FM and NM adjacent to the interface contribute to the SSE (see Fig. 1c). In addition, this planar architecture introduces operational anisotropy; the heat flux and magnetization of FM must be in the directions perpendicular and parallel to the FM/NM interface, respectively, thereby restricting practical applicability. To overcome these structural limitations and enable scalable SSE-based energy harvesting, a transition from thin-film architectures to bulk-compatible, three-dimensional geometries is essential, which we refer to as trans-scaling.

One approach to implementing trans-scaling is to engineer extrinsic device parameters. Specifically, a three-dimensional nanostructured composite material^24,43^ can be constructed by embedding multiple NM-coated FM domains throughout the bulk (Fig. 1b). This design effectively extends conventional quasi-two-dimensional SSE devices into bulk composites, increasing the usable thermal volume, which enhances the overall heat-to-electricity conversion capability without relying on high-resistance thin-film architectures. This concept, referred to as the trans-scale SSE, leverages composite architectures in which NM channels are distributed throughout the FM matrix, allowing volumetric utilization of spin currents and circumventing the intrinsic limitations imposed by *λ*_s_ and *λ*_m_ (Fig. 1d).

To elucidate the physical basis that enables the trans-scale SSE to survive such a geometrically complex FM/NM interface network, we first recall that in an FM/NM bilayer the ISHE field obeys

1 $${{{{\bf{E}}}}}_{{{{\rm{ISHE}}}}}\propto {{{{\bf{j}}}}}_{s}\times \hat{m}$$

where ${{{{\bf{j}}}}}_{s}$ is the spatial direction of the thermally driven spin current induced by a temperature gradient $\nabla T$, and $\hat{m}$ is the magnetization (spin-polarization) direction. For an arbitrarily oriented interface patch with unit normal $\hat{n}$, the injected spin current flows along $\hat{n}$, and its magnitude scales with the normal projection of $\nabla T$. The local ISHE response therefore generalizes to

2 $${{{{\bf{E}}}}}_{{{{\rm{ISHE}}}}}(\hat{n})\propto (\hat{n}\cdot \nabla T)(\hat{n}\times \hat{m}).$$

Because both prefactors in Eq. (2) reverse sign under inversion of the interface normal ($\hat{n}\to -\hat{n}$), their product remains invariant, ensuring that rough, concave, convex, or oppositely oriented facets contribute constructively rather than canceling. When the orientations of $\hat{n}$ are statistically isotropic in three-dimensional space, the orientational average reduces to

3 $$\left\langle (\hat{n}\cdot \nabla T)(\hat{n}\times \hat{m})\right\rangle=\frac{1}{3}(\nabla T\times \hat{m})$$

indicating that the emergent macroscopic SSE retains the usual ISHE symmetry $\nabla T\times \hat{m}$ independent of morphological complexity, with a geometric reduction factor of 1/3. A full derivation is provided in Supplementary Note 1.

Despite its conceptual promise, the realization and development of such three-dimensional SSE devices remain challenging due to difficulties in NM-FM coating and sintering. First, conventional methods for coating FM with NM, such as atomic layer deposition (ALD), rely on chemical precursors and often produce poor FM/NM interfacial quality due to residual organic contaminants and weak mechanical FM/NM bonding. These interfacial problems significantly degrade the spin mixing conductance; for example, YIG/Pt interfaces prepared by ALD exhibit spin mixing conductance reduced by a factor of ~20 compared to those prepared by sputtering^44^. FMMs, while easier to fabricate into NM microchannel structures, exhibit very short *λ*_s_ on the order of a few nanometers^42^ and are susceptible to oxidation, which limits their effectiveness in such composites^43^. Moreover, electrical shunting between the FMM and NM can substantially reduce the SSE signal generated in the NM layer. The anomalous Nernst effect (ANE)^10,45^ generated in the FMM can also interfere with the detection of the pure SSE contribution. Therefore, oxide-based FMIs with long *λ*_m_, such as YIG, are more desirable. However, they are structurally more difficult to process due to their thermodynamic stability, which requires high temperatures for sintering (e.g., above 900  °C for YIG^46^). Such high temperatures can lead to several issues in the thin NM layer^47,48^, including increased surface roughness, film discontinuity, and dewetting, all of which degrade layer quality (see Supplementary Note 2 for detailed discussion). Overcoming these challenges is thus crucial for realizing SSE-based energy harvesting devices that can effectively utilize heat from insulators at macroscopic scales. While the conceptual structure of bulk SSE composites was outlined nearly a decade ago^43^, their practical realization has remained difficult due to the intrinsic constraints discussed above. The successful demonstration of such systems would represent a significant advance toward practical spin-caloritronic applications.

## Results

### Fabrication of YIG-Pt composite materials

**Fig. 2:**
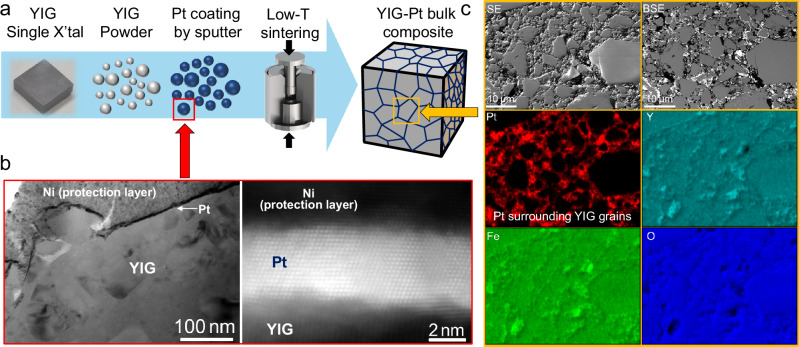
Fabrication and characterization of YIG-Pt composite materials for trans-scale spin Seebeck effect. **a** Schematic illustration of the fabrication process. YIG powders are coated with Pt using dynamic powder sputtering, followed by low-temperature sintering to produce bulk composites. **b** TEM images showing uniform nm-scale Pt coating on the surface of YIG powders. **c** Microstructural and compositional characterization of the bulk composite. SEM images (SE and BSE) reveal grain size distribution. EDX elemental mappings confirm uniform distribution of Y, Fe, and O, throughout the composite matrix and Pt surrounding YIG grains.

Here, we demonstrate the trans-scale SSE using FMI-NM composite materials composed of multiple YIG-Pt domains distributed throughout the bulk (Fig. 1b). To fabricate this structure, we established a dynamic powder sputtering system^49^ (Supplementary Fig. 1 for photographic setup) that enables uniform, nanometer-scale Pt coating on YIG powders without the use of chemical precursors (Fig. 2 and Methods). Uniform Pt coating was achieved by simultaneously rotating and vibrating the YIG powders during deposition. Figure 2b shows a transmission electron microscopy (TEM) image of Pt-coated YIG powders, confirming the formation of a continuous 5-nm-thick Pt layer. When the Pt thickness was reduced below 5 nm, the coating became non-uniform (Supplementary Fig. 2).

This metallic coating not only provides spin-caloritronic functionality (i.e., thermal spin pumping), but also improves mechanical adhesion between powders owing to the ductility and high atomic mobility of metals compared to oxides. As a result, the Pt-coated powders could be sintered through metal-mediated ductile channels at low temperatures, including 300 °C and even at room temperature (Methods), while maintaining the conducting paths in the bulk composite. In contrast, pure YIG powders could not be densified under the same conditions, highlighting the critical role of Pt layers in enabling densification.

Scanning electron microscope (SEM) images reveal the formation of micrometer-scale Pt channels, which increase the effective volume of both FM and NM materials contributing to the SSE (Fig. 2c). X-ray diffraction (XRD) confirmed that the YIG remains the predominant phase without the formation of secondary phases during fabrication (see Supplementary Note 3 and Supplementary Fig. 3 for detailed discussion). A more comprehensive discussion of potential contributions from secondary phases is presented in Discussion, where we also examine the exclusion of possible signal contamination from parasitic thermoelectric effects.

Four composite samples were prepared to experimentally demonstrate the trans-scale SSE. These samples differed in Pt thickness (15 nm and 30 nm), both of which are well above *λ*_s_ of Pt ( < 5 nm^35^), ensuring clear observation of the SSE without discontinuities in the Pt channels. In addition, two different sintering conditions were employed: pressing at 300 °C and room temperature under high pressure (Methods). The sintered pellets were labeled according to their Pt thickness and sintering temperature (i.e., 15-300, 15-RT, 30-300, and 30-RT), and they exhibited well-consolidated structures with relative densities ranging from 67% (15-RT) to 73–75% (15-300, 30-300, and 30-RT), as shown in Supplementary Fig. 4.

### Electrical and thermal conductivity of nanostructured bulk composites

We then evaluated the electrical conductivity (*σ*) of the bulk composite by normalizing the volumetric electrical conductance with the geometric dimensions of the composite, including YIG and Pt (Methods). The measured *σ* values range from (5–8) $\times$ 10^2 ^S/m for 15 nm Pt samples and (23–50) $\times$ 10^2 ^S/m for 30 nm Pt samples at room temperature (Supplementary Fig. 4a), all of which fall within the “bad metal” regime^50^. These values are higher than those of typical homogeneous semiconductors (non-doped Si ~ 1.5 $\times$ 10^−3 ^S/m and Ge ~ 2 S/m) and oxide-based bulk ANE materials ((3–26) × 10^2 ^S/m)^51–53^.

Next, we estimated the thermal conductivity (*κ*) by multiplying the measured thermal diffusivity, specific heat, and density (Supplementary Fig. 4). The *κ* values are 1.46–1.87 W/mK, which are substantially lower than those reported for polycrystalline YIG (~4.9 W/mK) and single-crystalline YIG (~7–8 W/mK)^46,54^. Such low *κ*, corresponding to 30–38% of the polycrystalline reference, cannot be attributed solely to the relative density, for example, 75% for 30-RT. The reduction likely reflects multiple phonon-scattering channels introduced by the composite structure, including porosity, grain boundaries, interface defects, and thermal boundary resistance at the YIG/Pt interfaces. In insulating YIG, heat conduction is mainly governed by phonons, with a relatively small magnon contribution expected at room temperature in polycrystalline samples^46,54^. Therefore, controlling the YIG/Pt interfacial density, defect structure, and pore morphology may provide an additional route for thermal-conductivity engineering in bulk SSE composites.

### Observation of trans-scale SSE in nanostructured bulk composites

**Fig. 3:**
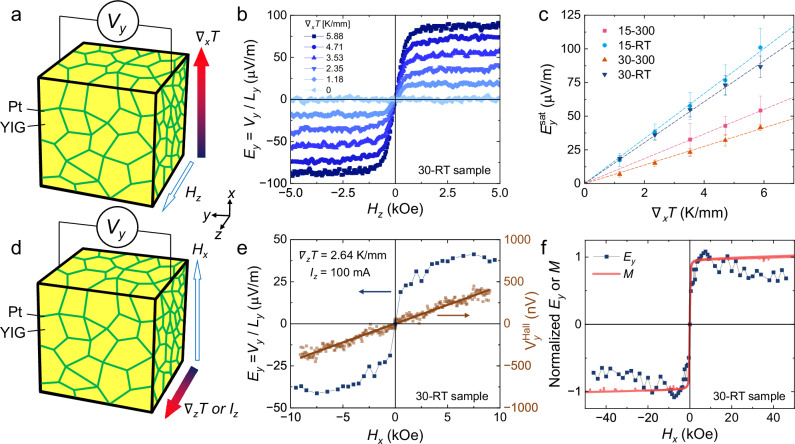
Observation of the trans-scale spin Seebeck effect. **a** Schematic illustrations of *H*_*z*_*-*${\nabla }_{x}T$ configuration. **b** Measured transverse electric field (*E*_*y*_) under $\nabla T$ in *H*_*z*_-${\nabla }_{x}T$ configuration as a function of *H*. The subscripts *x*, *y*, and *z* indicate the corresponding directions of the applied temperature gradient ($\nabla T$), charge current (*I*), and magnetic field (*H*). (c) Saturated electric field (${E}_{y}^{{{{\rm{Sat}}}}}$) plotted as a function of ${\nabla }_{x}T$. Error bars in (**c**) represent the standard deviation. **d** Schematic illustrations of *H*_*x*_-${\nabla }_{z}T$ configuration. **e** Measured transverse electric field (*E*_*y*_) under $\nabla T$ in *H*_*x*_-${\nabla }_{z}T$ configuration. The measured Hall voltage (${V}_{y}^{{{{\rm{Hall}}}}}$) is also presented for reference, along with a linear fitting curve. **f** Normalized *E*_*y*_ and Magnetization (*M*) of the 30-RT sample under high *H*. Detailed *M*-*H* curves for the other samples are provided in Supplementary Fig. 6.

We now present the observation of the trans-scale SSE in the YIG-Pt composites under two distinct magnetothermal configurations: dubbed “*H*_*z*_-${\nabla }_{x}T$” (“*H*_*x*_-${\nabla }_{z}T$”) configuration measuring voltage into the *y*-direction, while the orthogonal *H* is applied to *z* (*x*)-direction and temperature gradient ($\nabla$*T*) is applied to *x* (*z*)-direction, as shown in Fig. 3a, d). While rotating the magnetothermal configurations are not expected to generate a consistent SSE signal in conventional FM/NM planar film structures^14,20^ due to lack of spin pumping under out-of-plane *H*, we show that both geometries consistently produce SSE signals in our composites, highlighting a distinctive feature of isotropic trans-scale SSE. In the following, we provide a systematic evaluation to rigorously validate the observation by excluding potential parasitic contributions that could influence voltage measurement and by assessing the effects of secondary phases. These parasitic signals include the Seebeck effect driven by oblique heat flux^55,56^, the (proximity-induced) ANE^14,20,57^, and magnetoresistance effects.

We first focus on the observed transverse electric field (*E*_*y*_) in the *H*_*z*_-${\nabla }_{x}T$ configuration (Fig. 3a). The signal was symmetrized with respect to *H* to remove field-even components, such as the magneto-Seebeck effect caused by unintended transverse heat gradients (${\nabla }_{y}T$) in Pt. As shown in Fig. 3b, a clear field-odd signal was observed in the 30-RT sample within the *H*_*z*_ range of $\pm \,$5 kOe. The linear dependence of *E*_*y*_ on ${\nabla }_{x}T$ was confirmed in Fig. 3c, using ${E}_{y}^{{{{\rm{sat}}}}}$ denoting *E*_*y*_ at the saturated fields (>2.5 kOe). These results directly confirm that the observed signal originates from magnetism-induced transverse thermoelectric conversion, with longitudinal thermoelectric and magneto-electric contributions effectively excluded. The linear slope of Fig. 3c corresponds to the spin Seebeck coefficient (*S*_SSE_ $\equiv \,{E}_{y}^{{{{\rm{sat}}}}}/{\nabla }_{x}T$), shown in Supplementary Fig. 5. Samples sintered at room temperature exhibited *S*_SSE_ values of 17.3 nV/K (15-RT) and 14.8 nV/K (30-RT), which are higher than those of samples sintered at 300 °C (9.2 nV/K for 15-300 and 7.5 nV/K for 30-300). The 15-nm-thick Pt samples exhibited slightly higher *S*_SSE_ than the 30-nm-thick Pt samples, qualitatively consistent with the typical Pt thickness dependence of *S*_SSE_ in the conventional YIG/Pt systems due to the small *λ*_s_ of Pt^35^. Using the estimated *σ* and *S*_SSE_, we calculated the transverse power factor (PF_SSE_ = *σS*_SSE_^2^) driven by SSE. Supplementary Fig. 5b shows that 30-RT exhibited the largest PF_SSE_ (5.1 $\times$ 10^−13 ^W/mK^2^), approximately 630% higher than that of 15-300 (0.7 $\times$ 10^−13 ^W/mK^2^), strongly suggesting the importance of optimizing the Pt thickness and sintering conditions to enhance the transverse thermoelectric performance.

Next, we demonstrate the SSE in the *H*_*x*_*-*${\nabla }_{z}T$ configuration using the 30-RT sample by rotating it 90*°* to the original magnetothermal configuration, where the *E*_*y*_ signal was measured under *H*_*x*_ and ${\nabla }_{z}T$ (Fig. 3d). In conventional anisotropic FM/NM thin-film structures, no SSE signal is expected in this rotated magnetothermal configuration, due to the absence of spin pumping at the FM/NM interface. Only (proximity-induced) ANE can be captured in such cases^14,20^. Figure 3e shows a clear field-odd signal, with *S*_SSE_ measured as 14.4 nV/K, which is consistent with that obtained in the *H*_*z*_-${\nabla }_{x}T$ configuration (Fig. 3c and Supplementary Fig. 5). This consistency supports the assignment of the signal to SSE and demonstrates the reproducibility of the bulk SSE response under orthogonal magnetothermal configurations, while confirming the directional isotropy of the system and its potential for energy harvesting applications.

### Validation of trans-scale SSE and exclusion of parasitic effects

One may raise concerns about parasitic contributions from the (proximity-induced) ANE, which can arise in both *H*_*z*_*-*${\nabla }_{x}T$ and *H*_*x*_*-*${\nabla }_{z}T$ configurations, if secondary (proximity-induced) FMM phases were unintentionally formed during fabrication. However, we rule this out based on multiple lines of evidence. First, XRD measurements show no detectable formation of FMMs, such as Fe_3_O_4_ or FePt (Supplementary Fig. 3b and Supplementary Note 3), thereby excluding their contributions to ANE. This is further supported by the magnetization data: if such FM phases were present, the saturation magnetization would increase, considering that the ferrimagnetic YIG (37.9 emu/g) exhibits much lower values than those of common Fe-based FMs (85–92 emu/g for Fe_3_O_4_^58^, 74-76 emu/g for γ-Fe_2_O_3_^59^, and 186-218 emu/g for α-Fe^60^). In contrast, we observe a decrease in saturation magnetization upon the deposition of paramagnetic Pt (Supplementary Fig. 6), and the magnetization curve shows a single, well-defined hysteresis loop without anomalies, confirming the absence of secondary magnetic phases (Supplementary Fig. 6 for detailed low-field data).

We also exclude the contribution of proximity-induced ANE because the magnetic proximity effect between YIG and Pt and its contribution to transverse thermoelectric effect are well known to be negligibly small, as demonstrated by X-ray magnetic circular dichroism and transport studies^14,61–64^. Consistently, SQUID-VSM measurements show no additional magnetic component or anomalous magnetic behavior. Even if local strain-induced interfacial modification exists below the detection limit, it would affect interfacial spin transparency or SSE-driven ISHE efficiency^65^ rather than generate an independent ANE-like voltage. To further confirm the absence of such contributions from secondary (proximity-induced) FMMs, we performed Hall measurements, measuring *E*_*y*_ under a charge current *I*_*z*_ and *H*_*x*_, to sensitively detect any (proximity-induced) FMMs effectively contributing to the transport (Fig. 3e). The Hall voltage (${V}_{y}^{{{{\rm{Hall}}}}}$) shows a linear response to *H*_*x*_, indicating the absence of (proximity-induced) FMMs effectively contributing the transverse signals. Furthermore, the transverse thermoelectric signal exhibits a suppression at high magnetic fields (Fig. 3f), which is a well-established hallmark of the SSE^13,15^. This suppression arises from the field-induced reduction of thermally excited magnons and is not expected for ANE of FMM impurities, whose transverse voltage simply follows the magnetization and saturates at high fields. This high-field damping therefore provides an additional signature that the observed transverse voltage originates from magnon-driven SSE rather than ANE.

**Fig. 4:**
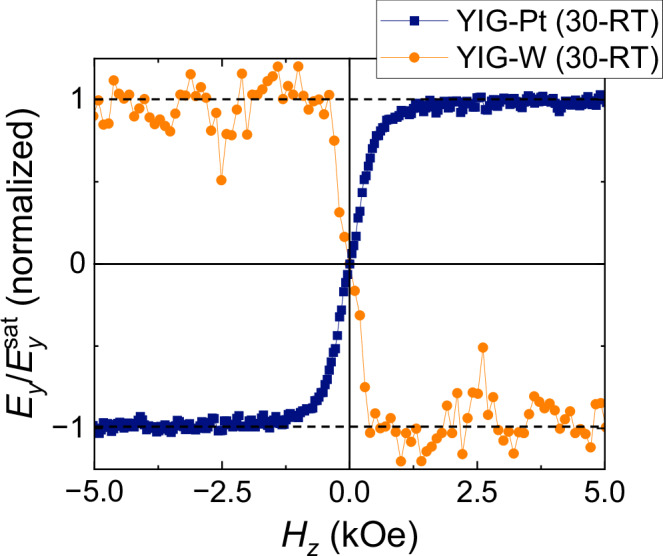
Normalized spin Seebeck voltage for YIG–Pt and YIG–W bulk composites. The two samples exhibit opposite signs of transverse thermoelectric voltage, reflecting the opposite signs of the spin Hall angles of the nonmagnetic metals, Pt (positive) and W (negative). The signal in the YIG–W composite appears noisier due to the large electrical resistance of the non-optimized composite. See Supplementary Note 4 for fabrication details and discussion.

Finally, to provide an independent and unambiguous confirmation, we prepared a YIG–W composite, where W has a negative spin Hall angle. Figure 4 shows that the transverse thermoelectric voltage of the YIG–W composite in the *H*_*z*_-${\nabla }_{x}T$ configuration exhibits a clear sign reversal relative to the YIG–Pt composite under otherwise identical conditions, despite the higher noise level associated with the larger electrical resistance of the control sample (Supplementary Note 4). This inversion provides direct evidence that the observed transverse voltage originates from the magnon-driven thermal spin pumping and its ISHE-driven spin-charge conversion in the bulk composite, establishing the trans-scale SSE.

We additionally note that the SSE originates from magnons generated in YIG, not from secondary magnetic phases, such as antiferromagnetic α-Fe_2_O_3_ and YFeO_3_. While both are reported to exhibit long-range magnon transport^66,67^, their formation could not be distinguished in the XRD data shown in Supplementary Fig. 3 and their magnon transport is known to be strongly anisotropic and to require high magnetic fields. These characteristics suggest that they are inert to magnon transport within the field range used in our measurements.

### Thickness-dependent power scaling enabled by volumetric spin conversion

**Fig. 5:**
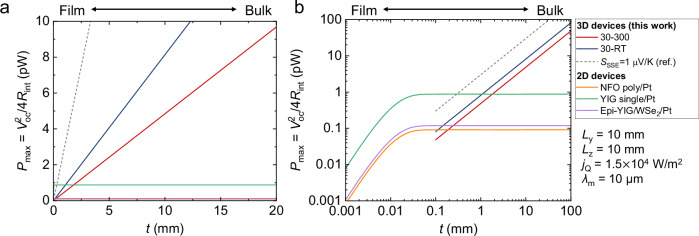
Thickness-dependent scaling of maximum output power in transverse spin Seebeck devices. **a** Linear-scale plot and **b** logarithmic-scale plot of the maximum output power *P*_max_ as a function of ferromagnetic layer thickness. *P*_max_ was calculated under identical device area (*L*_*y*_ = *L*_*z*_ = 10 mm) and heat-flux (*j*_Q_ = 1.5$\times$10^4 ^W/m^2^) conditions using ${P}_{\max }={V}_{{{{\rm{oc}}}}}^{2}/4{R}_{{{\mathrm{int}}}}$, where *V*_oc_ is the open circuit voltage and *R*_int_ is the internal resistance. A representative magnon diffusion length of *λ*_m_ = 10 µm was used for the 2D reference calculations. The 2D reference systems were modeled using representative material parameters from polycrystalline NiFe_2_O_4_ (NFO poly)/Pt^21^, single-crystalline YIG (YIG single)/Pt^69^, and epitaxial YIG films (Epi-YIG)/WSe_2_/Pt^34^ heterostructures reported in the literature. For the 3D architecture, the thickness-dependent scaling is shown only for thicknesses exceeding both *λ*_m_ and the experimentally accessible range (>0.1 mm), thereby representing the volumetric scaling regime. Detailed assumptions and parameter values are provided in Supplementary Note 5.

We evaluate the device-level performance using the maximum output power,

4 $${P}_{\max }=\frac{{V}_{{{{\rm{oc}}}}}^{2}}{4{R}_{{{\mathrm{int}}}}},$$

where *V*_oc_ is the open-circuit voltage and *R*_int_ is the internal resistance of the device. To isolate the architectural contribution, we fix the lateral device dimensions (*L*_*y*_ and *L*_*z*_) and the applied heat-flux boundary condition, while varying only the device or FM thickness *t* (Fig. 5).

For conventional quasi-two-dimensional interfacial SSE devices (Fig. 1a), the open-circuit voltage follows ${V}_{{{{\rm{oc}}}}}^{2{{{\rm{D}}}}}(t)\propto {S}_{0}(1-{e}^{-t/{\lambda }_{m}})$. Under fixed NM geometry, the internal resistance remains approximately thickness independent. Therefore,

5 $${P}_{\max }^{2{{{\rm{D}}}}}\left(t\right)\propto {(1-{e}^{-t/{\lambda }_{m}})}^{2},$$

which saturates for $t\gg {\lambda }_{{{{\rm{m}}}}}$. In the present analysis, a representative *λ*_m_ = 10 µm, as reported for YIG at room temperature^17,68^, was adopted for the 2D reference calculations.

In contrast, for the present quasi-three-dimensional composite, the percolated metallic NM network increases the effective conductive cross-section with thickness, resulting in ${R}_{{{\mathrm{int}}}}^{3{{{\rm{D}}}}}\propto 1/t$. Within the experimentally accessible thickness range ($\gg {\lambda }_{{{{\rm{m}}}}}$), the effective SSE is not limited by interfacial magnon diffusion and therefore does not exhibit the saturation characteristic of two-dimensional systems. Consequently,

6 $${P}_{\max }^{3{{{\rm{D}}}}}\left(t\right)\propto t,$$

leading to a non-saturating increase of output power with increasing thickness. A detailed derivation of these scaling relations, including expressions in terms of experimentally measurable parameters, is provided in Supplementary Note 5.

For the three-dimensional composite, experimentally measured transport parameters from the 30-300 and 30-RT samples were used for the scaling analysis, including *κ* = 1.87 W/mK, *σ* = 2.31 $\times$ 10^3 ^S/m, and *S*_SSE_ = 14.8 nV/K for the 30-RT sample. For the quasi-two-dimensional reference systems, representative material parameters reported for polycrystalline NiFe_2_O_4_/Pt^21^, single-crystalline YIG/Pt^69^, and epitaxial YIG films/WSe_2_/Pt^34^ heterostructures were adopted, using literature values of *S*_SSE_, *κ* of the FM layer and *σ* of the NM layer. Using these parameters (see Supplementary Table 1), Fig. 5 presents *P*_max_ as a function of thickness under identical device area (10 mm $\times$ 10 mm) and heat-flux (*j*_Q_ = 1.5 $\times$ 10^4 ^W/m^2^) boundary conditions. For the three-dimensional composite, the scaling analysis is shown only in the regime where the thickness exceeds both *λ*_m_ and the experimentally accessible range (>0.1 mm), corresponding to the volumetric scaling regime. The quasi-three-dimensional composite architecture exhibits an approximately linear increase in output power with thickness, whereas conventional quasi-two-dimensional devices exhibit saturation behavior governed by *λ*_m_. This contrast demonstrates that the present architecture removes the intrinsic interfacial thickness limitation characteristic of thin-film SSE systems and establishes a bulk-operating scaling regime enabled by volumetric spin-to-charge conversion. The dashed lines in Fig. 5 represent projected scaling behavior assuming higher intrinsic *S*_SSE_ comparable to those reported in optimized thin-film systems (on the order of 1 µV/K), while maintaining the same three-dimensional architecture and boundary conditions. Because the maximum output power in the volumetric regime follows ${P}_{\max }^{3{{{\rm{D}}}}}\propto {S}_{{{{\rm{SSE}}}}}^{2}t$, increasing the intrinsic *S*_SSE_ enhances the slope of the linear thickness scaling without altering the linear thickness dependence.

## Discussion

In summary, we have demonstrated the observation of trans-scale SSE using YIG-Pt bulk composite materials. The composites were fabricated via a dynamic powder sputtering method followed by low-temperature sintering. These bulk materials exhibit clear transverse thermoelectric signals at the bulk scale, consistent with volumetric spin-to-charge conversion. Through rigorous signal and material characterization, we confirm that the observed signals originate purely from the SSE of YIG, with negligible parasitic contributions.

We believe this study represents a key step toward the development of practical SSE-based thermoelectric devices by bridging the technical gap between conventional nanoscale SSE effects, which are limited by the spin and magnon diffusion lengths, and bulk device platforms. From the intrinsic (materials) perspective, further improvement in output power can be achieved by optimizing FM/NM interfaces^21,22^, for example by inserting topological materials^34,70^ or organic semiconductors^71^ at the interface, as well as engineering magnon transport in YIG independently from phonons^16,21^. In addition, replacing YIG with alternative high-performance FMI with lower Gilbert damping constants and longer magnon diffusion lengths, and adopting NM materials with a giant inverse spin Hall effect, such as topological insulators and Weyl semimetals^72–77^, could substantially increase conversion efficiency at bulk scale. These NM materials offer long spin diffusion lengths (up to 2 µm at low temperature^78,79^ and even at room temperature^80^) and spin Hall conductivities that exceed those of Pt by orders of magnitude, providing a direct route to boost spin Seebeck coefficient and the resulting output power. Many of these strategies have already been demonstrated independently in thin-film spintronics and spin caloritronics, where significant performance improvements compared to Pt-based systems have been achieved. Translating these advances into bulk architectures could therefore accelerate the development of practical SSE-based energy harvesters. Moreover, hybridization with other transverse thermoelectric phenomena, such as the ANE^7,10,43,81^, which has recently shown remarkable performance enhancements in topological materials^82–86^ and structurally heterogeneous systems^45,87–89^, may provide an additional route for enhancing bulk-scale performance. Hybrid transverse thermoelectric responses combining SSE and ANE have been reported in YIG/FMM bilayer systems, for example using Ni as the ferromagnetic metal^9^, where anomalous Nernst response and spin-to-charge conversion coexist within the same device geometry. In this context, employing FMMs that exhibit both a sizable ANE and efficient spin-to-charge conversion, including anomalous ISHE^90^, could enable constructive coexistence of ANE- and SSE-driven transverse voltages within the same device geometry. Because both effects share identical symmetry with respect to magnetization and thermal gradient, their contributions may, in principle, superpose and yield synergistic enhancement when their signs are aligned.

From the extrinsic (structural) perspective, our symmetry analysis in Eq. (3) reveals that the trans-scale SSE amplitude in the present composites contains a geometric reduction factor of 1/3 arising from the statistically isotropic distribution of local FMI/NM interface normals. Structural engineering that induces anisotropic or partially aligned interface orientations, for example, via controlled grain-shape anisotropy, magnetic-field-assisted forming, or directional sintering, can bias the distribution of interface normals and increase the geometric contribution toward the planar-film limit (i.e., the reduction factor is 1). This identifies structural orientation control as an additional, previously unexplored degree of freedom for amplifying trans-scale SSE signals in 3D architectures.

Altogether, the demonstration of trans-scale SSE in a bulk composite platform establishes a previously inaccessible regime of transverse thermoelectric conversion that extends well beyond the constraints of conventional thin-film architectures. By enabling volumetric spin-current generation and macroscopic ISHE conversion within a mechanically robust 3D network, this work provides a practical and scalable route for implementing spin-based thermoelectric functionality at bulk scale. Unlike interfacial thin-film SSE systems, the present architecture supports bulk-scalable geometries in which output power increases with device thickness without interfacial saturation, thereby expanding the accessible design space for transverse thermoelectric energy conversion. These results position trans-scale SSE composites as a promising platform for next-generation transverse thermoelectrics based on spin currents.

## Methods

### Sample preparation

The Pt-coated YIG powders were prepared using a customized dynamic sputtering system for powder coating (SUGA Co., Ltd., SSP-1500B) with a base pressure of less than 5 $\times$ 10^−4 ^Pa. The initial YIG powders were prepared by crushing YIG single crystals (Ferrisphere Inc.). The sputtering system vibrated the powders at a frequency of 22.5 Hz and rotated the powders at approximately 17 rpm to stir them. Pt was sputtered from a 99.99%-purity Pt target with an Ar gas flow of 6.0 sccm at room temperature. The plasma power was set to 50 W. The 5-nm Pt sample shown in Fig. 2 was deposited for 30 min, and the Pt thicknesses of the main samples (15 nm and 30 nm) were labeled based on the corresponding deposition durations.

The Pt-coated YIG powders were then sintered into bulk form by hot pressing under two low-temperature, high-pressure conditions: (1) 300 °C at 350 MPa and (2) room temperature at 500 MPa. Sintering was performed using a hot-pressing apparatus (AS ONE, H400-15) in air, employing a tungsten carbide die and punches with a diameter of 10 mm. The samples were cut using a diamond wire saw (Diamond WireTec, DWS.100) for subsequent materials characterization. All properties reported in this study were measured from samples prepared in a single batch.

### Structural characterization

Structural analysis was performed using a Rigaku MiniFlex600 X-ray diffractometer with Cr-Kα radiation (wavelength = 0.22897 nm). The diffraction angles were converted to commonly used Cu-Kα equivalent (wavelength = 0.15406 nm) for presentation in Supplementary Fig. 3. The X-ray tube was operated at 40 kV and 15 mA.

Microstructural observations were performed using a Carl Zeiss CrossBeam 1540EsB scanning electron microscope (SEM) equipped with energy-dispersive X-ray spectroscopy (EDS). Transmission electron microscopy (TEM) was carried out with a Titan G2 80-200 microscope equipped with a probe aberration corrector. TEM specimens were prepared using the lift-out technique with an FEI Helios 5UX dual-beam focused ion beam system.

### Measurement of thermal and electrical conductivities

The electrical conductivity (*σ*) was measured using a standard four-probe method with a current source (Keithley, 2450) and a nanovoltmeter (Keithley, 2182a). A linear voltage–current response was confirmed by measuring 9 points with reverse polarities of the current.

The thermal conductivity (*κ*) was estimated by multiplying thermal diffusivity, heat capacity, and density. Owing to the statistically random orientation of Pt-coated YIG particles in the sintered composite, the macroscopic thermal and spin transport properties are treated as effectively isotropic in the device-level analysis. The thermal diffusivity was obtained using a laser flash method (LINSEIS, LFA1000), specific heat was measured by DSC (Rigaku, Thermo plus EVO2), and density was calculated from the sample mass and volume at room temperature. The DSC measurement was conducted with an aluminum pan over a temperature range of 5 °C to 45 °C with a ramp rate of 1 °C/min. Alumina (Al_2_O_3_) powder was used as a reference for the DSC measurement.

### Measurement of the spin Seebeck effect

The SSE was measured in both *H*_*z*_*-*${\nabla }_{x}T$ and *H*_*x*_*-*${\nabla }_{z}T$ configurations. For *H*_*z*_*-*${\nabla }_{x}T$ configuration, two Cu wires were attached along the *y*-direction using silver epoxy and the sample was sandwiched between two thermally conductive, electrically insulating sapphire blocks serving as heat baths. Thermal grease was used at the interfaces to ensure a uniform temperature gradient. The *x*-directional temperature gradient applied was controlled using a PID-based temperature controller with measured temperature differences using differential thermocouples. It is worth noting that the measured temperature difference includes not only the drop across the sample but also the interfacial drops. This leads to an overestimation of the applied gradient across the samples and thus an underestimation of the reported spin Seebeck coefficients. Although this effect has been reported to be appreciable even at room temperature for YIG single crystals (despite the use of thermal grease)^91^, we expect the error here to be smaller because the thermal conductivity of our samples is lower (1.46–1.87 W/mK), i.e., about 18–27% of that of single-crystalline YIG (~7–8 W/mK^46,54^). Consequently, a larger fraction of the total temperature drop occurs within the sample rather than at the interfaces. A current source (ADVANTEST, R6243) and a multimeter (Keithley, 2000) were used for thermal gradient control. The voltage was measured using a nanovoltmeter (Keithley, 2182a) while applying magnetic fields with an electromagnet. The *H*_*x*_*-*${\nabla }_{z}T$ configuration was conducted in a similar manner, with the 30-RT sample bridged by two Cu blocks to apply a *z*-directional temperature gradient, as described in refs. ^87,92^. Adhesive thermal tapes (3 M, VHR0601-03) were used to electrically insulate the sample from the Cu blocks.

### Magnetic property analysis

The magnetic properties of the samples were investigated using a SQUID magnetometer (Quantum Design, MPMS). The measurements were conducted at *T* = 300 K with a sweep rate of 100 Oe/s. For the fine-scan measurement in Supplementary Fig. 6c, the magnetization was recorded at each field after stabilizing the magnetic field.

### Measurement of the Hall effect

The Hall effect shown in Fig. 3e was measured under *H*_*x*_ and *I*_*z*_ (same as the *H*_*x*_*-*${\nabla }_{z}T$ configuration) at room temperature using the DC resistivity option of a He cryostat (Cryogenic Limited, CFMS). Two electrodes were attached to the sample ends with silver epoxy to apply a current using a current source (Keithley, 2450) and two additional electrodes were attached orthogonally to capture the Hall voltage using a nanovoltmeter (Keithley, 2182a). The input current was 100 mA. The measurement was conducted over a magnetic field range of $\pm$10 kOe, consistent with the *H*_*x*_*-*${\nabla }_{z}T$ SSE measurement, using a sweep rate of 50 Oe/s. The data in Fig. 3e were symmetrized to remove the magnetic-field-even dependent components (e.g., magnetoresistance), following the same procedure used for the SSE data.

## Supplementary information

Supplementary Information

Transparent Peer Review file

## Source data

Source Data

## Data Availability

All data that support the findings of this study are available within the article and Supplementary Information. Source data underlying the plots in the main and Supplementary Figs. are provided with this paper as a Source Data file. Source data are provided with this paper.
