# Supplementary material for: Trans-scale spin Seebeck effect in nanostructured bulk composites based on magnetic insulator
Source: Nat Commun. 2026 Jul 21;17:6389. doi: 10.1038/s41467-026-75232-0 (PMC13389295; doi:10.1038/s41467-026-75232-0)
Supplement: Supplementary file 1 — Supplementary Information [file 41467_2026_75232_MOESM1_ESM.pdf]

Supplementary Information of

Trans-scale spin Seebeck effect in nanostructured bulk composites based on magnetic insulator

Sang J. Park[1,*], Keisuke Hirata[2,3], Hossein Sepehri-Amin[1], Fuyuki Ando[1], Takamasa Hirai[1], and Ken-ichi Uchida[1,3,*]

[1] National Institute for Materials Science, Tsukuba 305-0047, Japan

[2] Toyota Technological Institute, Nagoya 468-8511, Japan

[3] Department of Advanced Materials Science, Graduate School of Frontier Sciences, The University of Tokyo, Kashiwa 277-8561, Japan

*Correspondence to: PARK.SangJun@nims.go.jp (S.J.P.); UCHIDA.Kenichi@nims.go.jp (K.U.)

Table of contents

1. Supplementary Notes 1–5 p.3–8

2. Supplementary Figures 1–6 p.9–12

3. Supplementary Table 1 p.13

4. Supplementary References p.14

Supplementary Notes

Supplementary Note 1. Symmetry-based derivation of macroscopic ISHE field

Here we provide a brief derivation of Equations (2)–(3) in the main text, showing how a finite macroscopic inverse spin Hall effect (ISHE) field arises in a ferromagnetic material (FM)/normal metal (NM) composite with randomly oriented interface facets.

Local ISHE response for an arbitrary interface

For a local FM/NM interface patch with unit normal $\hat{n}$, the spin Seebeck effect (SSE) pumps a spin current perpendicular to the interface. Its local density can be expressed as

$$\mathbf{j}_s = j_0(\hat{n} \cdot \nabla T)\hat{n} \tag{S1}$$

where ∇T is the applied temperature gradient and $(\hat{n} \cdot \nabla T)$ selects its normal component. The ISHE converts this spin current into a transverse electric field,

$$\mathbf{E}_{\mathrm{ISHE}}(\hat{n}) \propto \mathbf{j}_s(\hat{n}) \times \hat{m} = (\hat{n} \cdot \nabla T)(\hat{n} \times \hat{m}) \tag{S2}$$

with the magnetization direction $\hat{m}$. Equation (S2) reproduces Eq. (2) in the main text. Under inversion of the interface normal ($\hat{n} \rightarrow -\hat{n}$), both factors in Eq. (S2) change sign,

$$(\hat{n} \cdot \nabla T) \rightarrow -(\hat{n} \cdot \nabla T), \qquad (\hat{n} \times \hat{m}) \rightarrow -(\hat{n} \times \hat{m}) \tag{S3}$$

so their product, and thus the local ISHE field, remains unchanged:

$$\mathbf{E}_{\mathrm{ISHE}}(-\hat{n}) = \mathbf{E}_{\mathrm{ISHE}}(\hat{n}). \tag{S4}$$

This invariance explains why concave, convex, rough, or oppositely oriented interface facets contribute with the same sign.

Orientational average for an isotropic interface network

In a polycrystalline bulk composite, the FM/NM interfaces are oriented in all directions. For such an isotropic distribution of interface normals, the average products of their components along two spatial directions (i.e., $n_i n_j$) must reflect this symmetry. As a result, the orientational average takes the rotationally symmetric form

$$\langle n_i n_j \rangle = \frac{1}{3}\delta_{ij} \tag{S5}$$

meaning that the components of $\hat{n}$ are equally sampled in all directions. Averaging the local response in Equation (S2) using Equation (S5) yields

$$\langle \mathbf{E}_{\mathrm{ISHE}} \rangle \propto \frac{1}{3}(\nabla T \times \hat{m}) \tag{S6}$$

which is Equation (3) in the main text. This result shows that even in a geometrically complex FM/NM composite, the macroscopic SSE preserves the usual ISHE symmetry $\nabla T \times \hat{m}$ while being reduced only by a geometric factor of 1/3 due to isotropic averaging.

Supplementary Note 2. Discussion on low-temperature sintering of YIG-Pt composites

Here, we discuss the low-temperature sintering of YIG-Pt composites. As stated in the main text, oxide YIG powders require high temperatures for sintering, typically over 900 ℃ due to their thermodynamically stable crystal structures and strong ionic and covalent bonding[1]. However, such high-temperature processing can lead to degradation of the thin Pt layers, including increased surface roughness, dewetting, and recrystallization into nanoparticles to minimize surface free energy. For example, Golosov et al.[2] reported the formation of hillocks several hundred nanometers high upon annealing sputter-deposited Pt-based multilayers at temperatures as low as 400 ℃, which correlated with changes in electrical resistivity. Similarly, Sui et al.[3] observed that annealing 10-nm-thick sputter-deposited Pt layers at 550 ℃ led to agglomerated nanostructures with significantly increased surface roughness and changed optical properties.

At the initial stage of the study, we performed high-temperature sintering of YIG-Pt powders at 800 °C, a temperature slightly lower than that reported in [1], to obtain high-density nanostructured composite samples. Although the resulting pellet exhibited a well-densified structure, it showed extremely high electrical resistance, exceeding the measurement range of our system (> MΩ), consistent with the expected degradation of thin Pt layer quality.

We therefore fabricated the YIG-Pt bulk pellets at low temperatures (300 °C and room temperature) under high pressure conditions, as described in the main text (Methods). The density of the samples reached up to 75% (30-RT sample, Supplementary Fig. 4d) relative to the YIG single crystal (5.11 g/cm^3). The low-temperature sintering was enabled by the surface coating of ductile metallic Pt layers. As a result, the Pt-coated YIG powders were more favorably pelletized through the Pt channels, providing mechanical adhesion between the powders. We also pelletized the YIG powders using the same conditions, intended as a control sample. However, we obtained a sample with poor mechanical robustness, exhibiting many macroscale cracks, suggesting the critical role of Pt coating for sintering.

In summary, the Pt coating of oxide YIG powders provides additional mechanical robustness to the system, allowing for low-temperature sintering.

Supplementary Note 3. Phase analysis based on XRD

We discuss potential structural changes during the deposition and sintering processes. θ-2θ powder XRD measurements were conducted using a Cr-Kα beam (wavelength = 0.22897 nm, Methods). The 2θ values in Supplementary Fig. 3 were converted to equivalent values based on the commonly used Cu-Kα radiation (wavelength = 0.15406 nm) for easier comparison.

The XRD pattern of the initial YIG powders, obtained by crushing YIG single crystals, is shown in Supplementary Fig. 3a. The predominant phase was identified as YIG, in agreement with the reference peaks of YIG (PDF#43-0507), without evidence of preferred crystal orientation. Only negligibly small peaks corresponding to Y_2O_3 (PDF#43-0661) were observed near $2\theta = 29°$. The XRD data from the main composite samples (15-300, 15-RT, 30-300, and 30-RT) consistently exhibited this small peak. The only noticeable difference between the composite samples and the initial YIG powder was the presence of broad Pt peaks (PDF#04-0802) near 2θ of 40° and 46°, indicative of low-crystallinity Pt formed on YIG. These results suggest that neither the room-temperature Pt sputtering nor the low-temperature sintering introduced additional secondary phases detectable by XRD.

To further exclude the formation of secondary magnetic phases, we replotted the XRD data (Supplementary Fig. 3a) with reference peaks of Fe- and Pt-based ferromagnetic materials (Fe_3O_4: PDF#19-0629 and FePt: PDF#43-1359), as shown in Supplementary Fig. 3b. These materials are known to contribute to transverse thermoelectric signals via the anomalous Nernst effect, which can be captured in both in-plane (Fig. 3a) and out-of-plane (Fig. 3d) magnetization configurations in the main text. However, no identifiable peaks corresponding to these phases were observed, confirming the absence of contamination in the observed trans-scale SSE signals shown in Fig. 3 of the main text.

Supplementary Note 4. Observation of trans-scale SSE in YIG–W bulk composite

To unambiguously verify the origin of the transverse thermoelectric voltage observed in the YIG–Pt composites, we performed a control experiment by replacing Pt with W, which has a negative spin Hall angle. The YIG–W composite was fabricated using the same procedure as the YIG–Pt samples (specifically the 30-RT condition). W was deposited onto the surface of YIG powders using the powder sputtering system and subsequently consolidated by high-pressure pressing at 500 MPa, identical to the procedure used for the Pt-based composite. The resulting YIG–W composite exhibited higher mechanical brittleness than the YIG–Pt samples, possibly due to the non-optimized fabrication conditions and/or partial oxidation of the W shells. The electrical resistance of the sample exceeded 10 kΩ, more than four orders of magnitude higher than that of the YIG–Pt composite with similar dimensions, suggesting that the W network was not fully continuous throughout the bulk. Further optimization may be required to obtain mechanically robust, electrically connected YIG–W composites. Due to the mechanical brittleness of the YIG–W composite, the samples could not be cut into well-defined rectangular geometries. Measurements were therefore performed using irregular pellet-like pieces, which makes absolute normalization by geometric dimensions challenging. To allow a meaningful comparison with the YIG–Pt results, the data presented in Fig. 4 of the main text are normalized to their maximum values. Despite these non-idealities, we clearly observed a transverse thermoelectric voltage with the opposite sign compared with the YIG–Pt composite, as shown in Fig. 4 in the main text. Although the signal is noisier possibly due to the high resistance of the sample, the sign reversal is evident and exceeds the fluctuation level. This behavior is fully consistent with the expected sign change of the spin Hall angle when replacing Pt (positive) with W (negative), thereby providing direct and independent evidence that the transverse thermoelectric voltage originates from magnon-driven thermal spin pumping in YIG and the spin-charge conversion via the ISHE in the NM.

Supplementary Note 5. Thickness-dependent scaling of output power in 2D and 3D spin Seebeck devices

General expression for output power

The transverse electric field generated by the SSE is expressed as

$$E_y = S_{\mathrm{SSE}} \nabla T, \tag{S7}$$

where S_{SSE} is the spin Seebeck coefficient and ∇T is the applied temperature gradient. The open-circuit voltage measured along the device length L_y is therefore

$$V_{\mathrm{oc}} = S_{\mathrm{SSE}} \nabla T L_y. \tag{S8}$$

When the device is connected to an external load resistance R_L, the output power is

$$P(R_L) = \frac{V_{\mathrm{oc}}^2 R_L}{(R_{\mathrm{int}}+R_L)^2}, \tag{S9}$$

where R_{int} is the internal resistance of the device. Maximum power transfer occurs when $R_L = R_{\mathrm{int}}$, yielding

$$P_{\max} = \frac{V_{\mathrm{oc}}^2}{4R_{\mathrm{int}}}. \tag{S10}$$

This expression forms the basis for the thickness-dependent scaling analysis presented below.

Thickness dependence in conventional quasi-two-dimensional SSE systems

In conventional quasi-two-dimensional SSE devices, spin-current generation and spin-to-charge conversion are interfacial processes. The S_{SSE} therefore depends on the magnetic layer thickness t as

$$S_{\mathrm{SSE}}(t) = S_0(1 - e^{-t/\lambda_m}), \tag{S11}$$

where S_0 is the bulk spin Seebeck coefficient and λ_m is the magnon diffusion length.

The open-circuit voltage becomes

$$V_{\mathrm{oc}}^{2\mathrm{D}}(t) = S_0(1 - e^{-t/\lambda_m}) \nabla T L_y. \tag{S12}$$

Under fixed normal-metal geometry, the internal resistance is approximately independent of the magnetic layer thickness ($R_{\mathrm{int}}^{2\mathrm{D}} \approx \mathrm{const.}$).

Accordingly, the maximum output power follows

$$P_{\max}^{2\mathrm{D}}(t) = \frac{[S_0\left(1-e^{-\frac{t}{\lambda_m}}\right)\nabla T L_y]^2}{4R_{\mathrm{int}}^{2\mathrm{D}}} \propto (1 - e^{-t/\lambda_m})^2. \tag{S13}$$

As t exceeds λ_m, both the voltage and the output power approach saturation. In the present scaling analysis, we adopt a representative magnon diffusion length of $\lambda_m \sim 10$ µm at room temperature as reported in the literature[4]. We note that additional magnon energy-relaxation mechanisms, which have been discussed at shorter length scales on the order of ~250 nm, are not included in this simplified model. Since the thickness range considered in this work

significantly exceeds the sub-micron regime, the diffusion-limited description provides an appropriate approximation for the purpose of architectural scaling comparison.

Thickness dependence in quasi-three-dimensional volumetric SSE composites

In the present three-dimensional composite architecture, spin-to-charge conversion occurs throughout the bulk volume via a percolated metallic network.

Under fixed lateral dimensions L_y and L_z, increasing thickness t increases the effective conductive cross-section of the interconnected metallic pathways. Using σ, the internal resistance scales as

$$R_{\mathrm{int}}^{\mathrm{3D}} \approx \frac{1}{\sigma}\frac{L_y}{L_z t} \propto \frac{1}{t}. \tag{S14}$$

Within the experimentally relevant thickness range (> 0.1 mm), the S_{SSE} does not exhibit the interfacial saturation characteristic of quasi-two-dimensional systems. The open-circuit voltage can therefore be expressed as

$$V_{\mathrm{oc}}^{\mathrm{3D}} = S_{\mathrm{SSE}}^{\mathrm{3D}} \nabla T L_y, \tag{S15}$$

where $S_{\mathrm{SSE}}^{\mathrm{3D}}$ is approximately thickness-independent in the absence of interfacial limitation.

Substituting into the general power expression yields

$$P_{\max}^{\mathrm{3D}}(t) = \frac{[S_{\mathrm{SSE}}^{\mathrm{3D}} \nabla T L_y]^2}{4R_{\mathrm{int}}^{\mathrm{3D}}} \propto t. \tag{S16}$$

Thus, in contrast to the saturation behavior of 2D interfacial SSE systems, the three-dimensional composite architecture exhibits a non-saturating power scaling with thickness within the accessible regime.

Architectural origin of the scaling difference

The key distinction between the two architectures arises from the spatial distribution of spin-to-charge conversion:

- In two-dimensional systems, conversion is confined to a thin interfacial region, leading to intrinsic thickness saturation governed by the magnon diffusion length.
- In the three-dimensional composite, conversion occurs throughout the bulk volume, while the internal resistance decreases with increasing conductive cross-section.

This difference in conversion topology results in fundamentally different thickness-scaling behaviors of output power.

Supplementary Figures

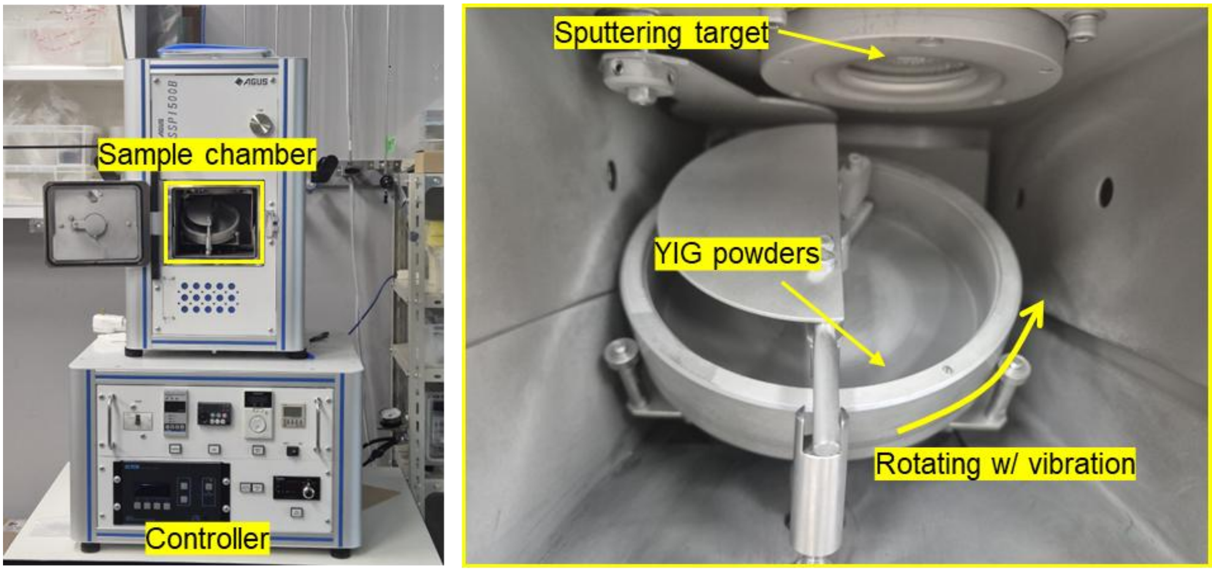

Supplementary Fig. 1| Experimental setup for dynamic powder sputtering.

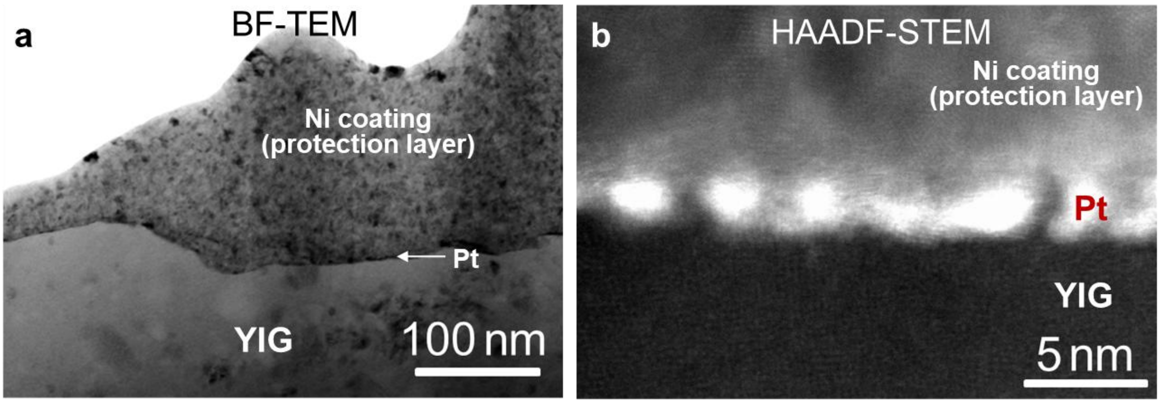

Supplementary Fig. 2| Transmission electron microscopy (TEM) results for 2.5-nm-thick Pt-deposited YIG powders. (a) Bright-field (BF) TEM image and (b) high angle annular dark-field scanning TEM (HAADF-STEM) image revealing island-like growth of Pt. Ni layers were deposited as a surface protective layer for TEM specimen preparation.

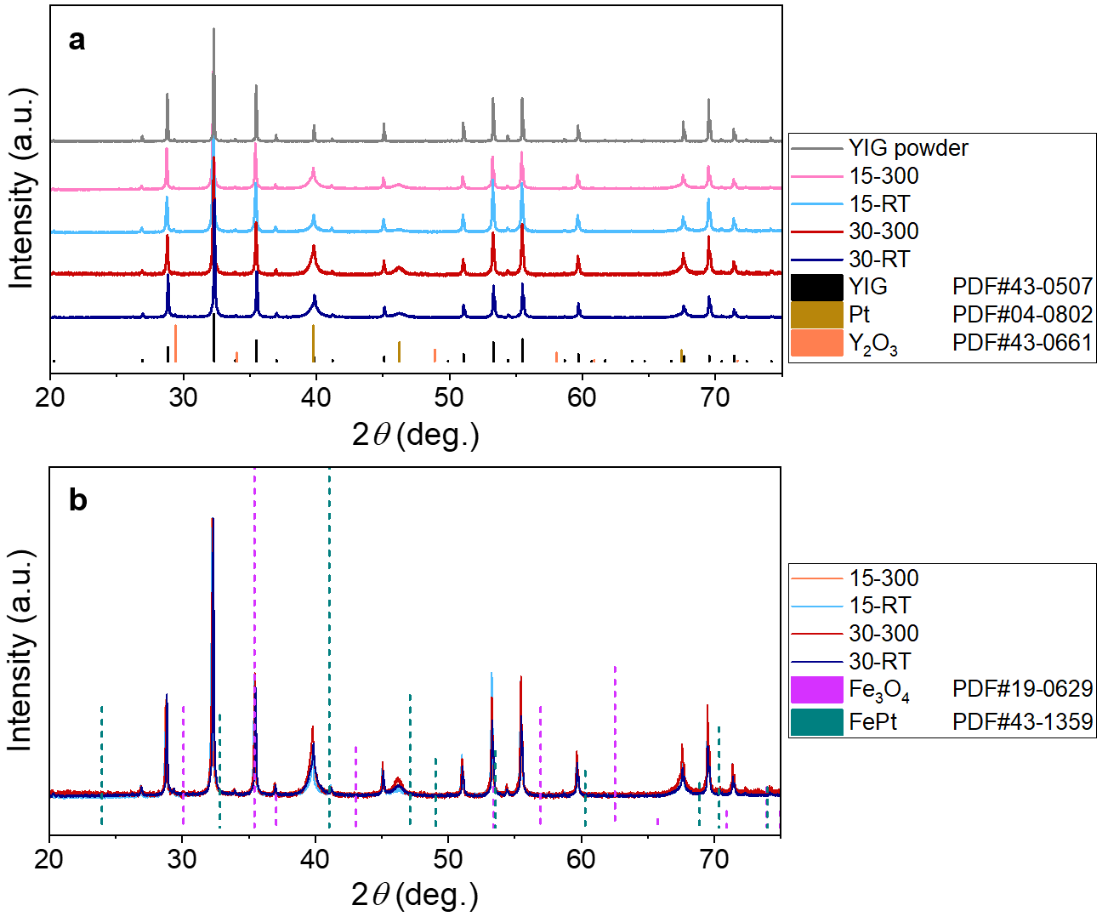

Supplementary Fig. 3| X-ray diffraction (XRD) analysis of YIG-Pt composite samples. (a) Comparison of XRD patterns for our samples with reference patterns of relevant constituent phases, including YIG, Pt, and Y_2O_3. (b) Examination of possible ferromagnetic impurity phases, such as Fe_3O_4 and FePt, which could contribute to the transverse thermoelectric signals.

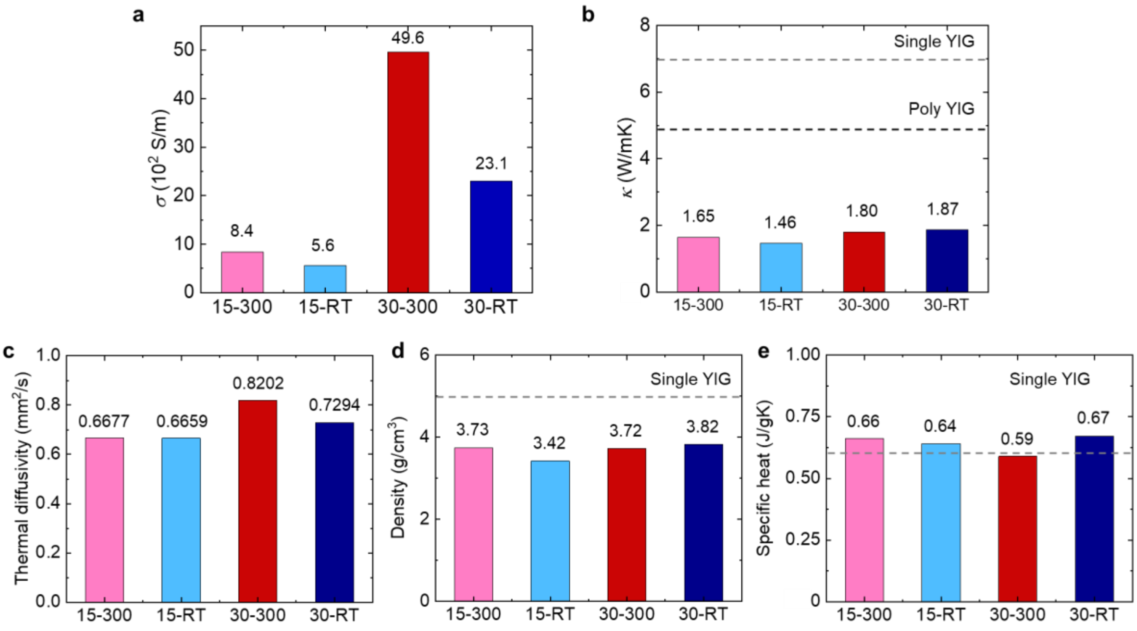

Supplementary Fig. 4| Transport and thermal properties of YIG-Pt composite samples. (a) Electrical conductivity (σ), (b) thermal conductivity (κ), (c) thermal diffusivity, (d) density, and (e) specific heat. The horizontal dashed lines indicate the material properties of single-crystalline or polycrystalline YIGs. The data in (a), (c)-(e) were experimentally measured, including those for the single crystal. In panel (b), κ values for single-crystalline and polycrystalline YIGs are literature values from [1], shown for comparison.

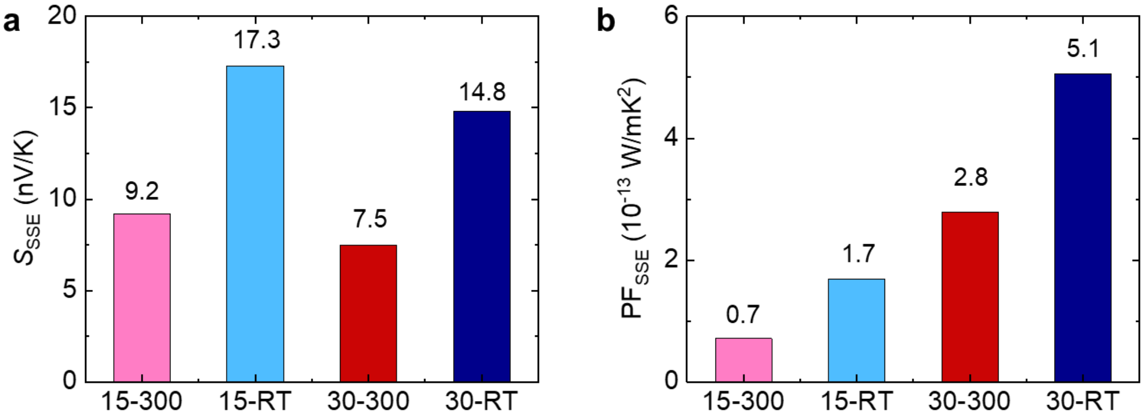

Supplementary Fig. 5| Transverse thermoelectric properties of the samples. (a) Spin Seebeck coefficient (S_{SSE}) and (b) transverse power factor ($PF_{SSE}= \sigma S_{SSE}^2$).

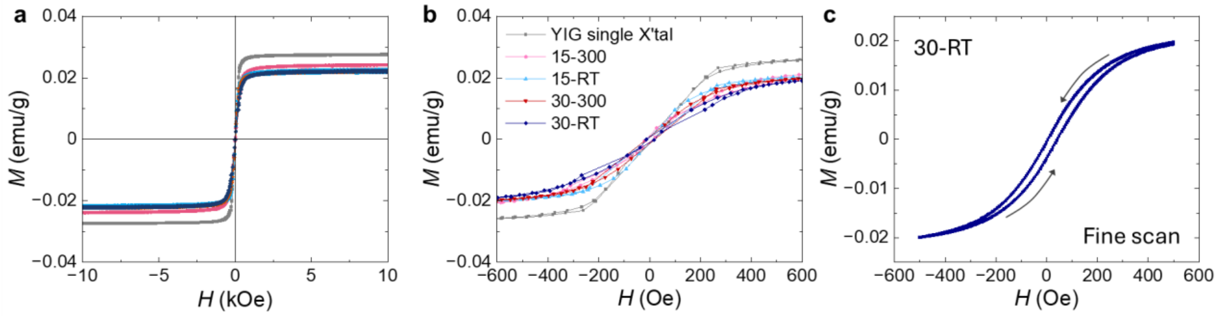

Supplementary Fig. 6| Magnetization curves of the samples. (a) High-field and (b) low-field magnetization data of the samples. (c) High-resolution magnetization curve of the 30-RT sample obtained with fine field steps within the low field range.

Supplementary Table

Supplementary Table 1. Material geometries and properties used for the maximum output power calculation. The materials include polycrystalline $NiFe_2O_4$ (NFO), single-crystalline YIG, YIG film grown by liquid-phase epitaxy (Epi-YIG). Blanks (-) indicate properties that are not applicable.

Ref.	Material			Thickness			σ of metallic layers (S/m)	κ (W/mK)	S_{SSE} (nV/K)
	Substrate	FM	NM	Substrate (mm)	FM (mm)	NM (nm)			
[5]	-	NFO polycrystal	Pt	-	0.5	10	1.60×10^6	10.37	330
[6]	-	YIG single crystal	Pt	-	1	10	5.30×10^6	7.9	428
[7]	GGG	Epi-YIG	WSe_2/Pt	0.5	0.005	5.7	1.46×10^6	7.8	392
This work	-	YIG polycrystal	Pt	-	~ 1 mm		4.96×10^3 (30-300)	1.80	7.5
							2.31×10^3 (30-RT)	1.87	14.8

Supplementary References

1. Miura, A. *et al.* Probing length-scale separation of thermal and spin currents by nanostructuring YIG. *Phys. Rev. Mater.* **1**, 014601 (2017).

2. Golosov, D. A. *et al.* Stability of the platinum electrode during high temperature annealing. *Thin Solid Films* **661**, 53–59 (2018).

3. Sui, M. *et al.* Effects of annealing temperature and duration on the morphological and optical evolution of self-Assembled Pt nanostructures on c-plane sapphire. *PLoS One* **12**, e0177048 (2017).

4. Cornelissen, L. J., Liu, J., Duine, R. A., Youssef, J. Ben & Van Wees, B. J. Long-distance transport of magnon spin information in a magnetic insulator at room temperature. *Nat. Phys.* **11**, 1022–1026 (2015).

5. Kim, M. Y., Park, S. J., Kim, G. Y., Choi, S. Y. & Jin, H. Designing efficient spin Seebeck-based thermoelectric devices via simultaneous optimization of bulk and interface properties. *Energy Environ. Sci.* **14**, 3480–3491 (2021).

6. Uchida, K., Kikkawa, T., Miura, A., Shiomi, J. & Saitoh, E. Quantitative temperature dependence of longitudinal spin Seebeck effect at high temperatures. *Phys. Rev. X* **4**, 041023 (2014).

7. Lee, S. K. *et al.* Enhanced Spin Seebeck Effect in Monolayer Tungsten Diselenide Due to Strong Spin Current Injection at Interface. *Adv. Funct. Mater.* **30**, 2003192 (2020).
