# Supplementary material for: Trans-scale spin Seebeck effect in nanostructured bulk composites based on magnetic insulator
Source: Nat Commun. 2026 Jul 21;17:6389. doi: 10.1038/s41467-026-75232-0 (PMC13389295; doi:10.1038/s41467-026-75232-0)
Supplement: Supplementary file 2 — Transparent Peer Review file [file 41467_2026_75232_MOESM2_ESM.pdf]

nature portfolio

Peer Review File

Trans-scale spin Seebeck effect in nanostructured bulk composites based on magnetic insulator

Corresponding Author: Dr Sang Park

This file contains all reviewer reports in order by version, followed by all author rebuttals in order by version.

Version 0:

Reviewer comments:

Reviewer #1

(Remarks to the Author)
The manuscript reports a bulk, nanostructured Pt/YIG composite that exhibits isotropic, volumetric spin Seebeck effect (SSE). The authors fabricated conformal Pt shells on YIG powders by dynamic powder sputtering and then densified the powders by low-temperature, high-pressure hot pressing to create a three-dimensional network of Pt channels embedded in an insulating magnetic matrix. This architecture overcomes the thin-film constraint imposed by short electronic spin-diffusion length and finite magnon diffusion length, enabling SSE signals in two orthogonal magnetothermal geometries. The composite simultaneously attains much higher effective electrical conductivity than conventional YIG/Pt thin films while maintaining low thermal conductivity. Parasitic thermoelectric contributions are carefully minimized by field-odd symmetrization, structural/magnetic phase checks, and linear Hall responses.

The study addresses a central bottleneck of SSE devices, namely, thin-film geometry and interfacial area limitation, by scaling the conversion process from “interfacial” to “volumetric” in a mechanically robust bulk composite. The device concept, materials processing, and measurements are all convincing.
This work clearly merits publication in Nature Communications. I therefore recommend acceptance after a single, focused minor revision that will substantially improve mechanistic clarity for readers.

It would greatly benefit readers if the manuscript explicitly stated the symmetry-based reasoning that explains why local interfacial contributions add constructively, even in a complex Pt/YIG morphology, and why the macroscopic SSE remains aligned with $\nabla T\times\mathbf{m}$. My understanding is as follows:

1. For a local interfacial patch with unit normal $\hat{n}$ (pointing from YIG to Pt), the heat-driven spin injection across the interface scales with the normal component of the temperature gradient:
$j_s^{int}(\hat{n}) \propto \hat{n} \cdot \nabla T$.

2. Inside Pt, the inverse spin Hall effect (ISHE) converts this spin current (flow direction $\parallel \hat{n}$) with spin polarization $\hat{m}$ into a local electric field:
$E_{ISHE} \propto \theta_{SH} (\hat{n} \times \hat{m})(\hat{n} \cdot \nabla T)$.

3. Crucially, under the inversion of the local normal $\hat{n} \to -\hat{n}$, both factors flip sign, and thus, their product does not. Hence, inner/outer facets of a Pt shell and rough/concave portions of the interface contribute with the same sign; they do not cancel.

4. If interface orientations are statistically isotropic, the orientational average leads to a simple geometric factor (second-moment identity $\langle n_i n_j\rangle = \delta_{ij}/3$):
$\langle(\hat{n} \times \hat{m}) (\hat{n} \cdot \nabla T) \rangle= (1/3) (\nabla T \times \hat{m})$.
This shows that the macroscopic electric field is aligned with ($\nabla T \times \hat{m}$) and is only reduced by a scalar factor of 1/3, explaining why the volumetric contributions are additive even throughout a random network.

If my understanding above is inaccurate in any respect, I would ask the authors to correct it and briefly state the precise mechanism. Otherwise, I suggest that the authors include a short explanation of this reasoning, either in the main text near Fig. 1 or in the supplementary information. This would neatly bridge the conceptual gap from Fig. 1c to Fig. 1d.

Reviewer #2

(Remarks to the Author)
The concept of bulk spin Seebeck composites has been reported in Ref. 43 Nat Commun 7, 13714 (2016), and thus it is not very new. This paper shows transverse thermoelectric voltages in Pt-YIG bulk composites. However, the spin Seebeck effect cannot be concluded by the results only with Pt. This paper does not show significant advance in physics understanding and clear advantage in performance. I cannot recommend the publication in Nature Communications. Please find my comments below.

1) This paper shows transverse thermoelectric voltages only in Pt-YIG bulk composites in which the spin Hall angle of Pt is positive. The transverse thermoelectric voltages could be anomalous Nernst effect in small amount of conductive Pt-doped YIG which is not detectable by XRD. To conclude the spin Seebeck and inverse spin Hall effects, the results should be also shown in the YIG bulk composites with nonmagnetic metal of negative spin Hall angle such as Ta and W.

2) The spin Seebeck coefficient is 17 nV/K in the Pt-YIG bulk composites, which is 30 times smaller than that of the conventional Pt/YIG layered structure (about 0.5 μV/K). The bulk composites do not show advantage in the thermoelectric coefficient.

3) In Table S1, what differences are the devices with the four effective electrical conductivities (σ_{eff}) for this work ? It is not meaningful to show the “effective” electrical conductivity of Pt/YIG by normalizing with the large geometric dimensions of insulating YIG. In fact, the electrical conductivity of the bulk composite is as poor as 10^2 S/m, which is 4 orders smaller than the electrical conductivity of the Pt layer 10^6 S/m.

Version 1:

Reviewer comments:

Reviewer #1

(Remarks to the Author)
I have reviewed the revised manuscript and the authors' response. The explicit inclusion of the symmetry-based reasoning for the constructive addition of local ISHE fields, together with the detailed derivation in the main text and Supplementary Note 1, has clarified the mechanism and fully addressed my concerns. I recommend this manuscript for publication in Nature Communications.

Reviewer #2

(Remarks to the Author)
1) As the authors’ response, the concept of bulk spin Seebeck composites is not new. The results are the spin Seebeck effect based on magnetic insulator which has been intensively studied. Again, this paper does not show significant advance in physics understanding.

2) The signal in Fig. 3f is noisy at high field for the YIG-Pt bulk composite. The signal of YIG-W bulk composite in Fig. 4 is also noisy. Why not show Ey for these results ? The performance of bulk spin Seebeck composite is really poor in this work.

3) I cannot find the authors’ response on the differences in the devices with the four effective electrical conductivities (σ_{eff}) from 5 x 10^2 S/m to 49 x 10^2 S/m for this work in Table S1. It is strange to compare the “effective” electrical conductivity of Pt/YIG by normalizing with the large geometric dimensions of insulating YIG.

In all, there is neither novel physics nor good performance of the material in this work. This paper does not meet the criteria of Nature Communications.

Reviewer #3

(Remarks to the Author)
The manuscript demonstrates the use of a unique powder sputter coating and sintering system that allows for nanoscale metallic networks to formed in a 3D FMI/NM composite material. This turns thermoelectric power conversion from the spin Seebeck effect from a 2D process to a 3D process, which is fundamentally more compatible with applications. I believe this work represents a significant technical feat by the authors, and is an genuine advance to the field. However, I do have some concerns as outlined below:

1. It is not clear to me that the performance of the 3D composite is significantly better than either conventional thermoelectrics, or 2D SSE thermoelectric systems. Authors point to the enhanced electrical conductivity compared to 2D SSE implementations, but this is only when an "effective" conductivity is used. This effective conductivity is misleading, since in the 2D implementation it counts the mm-scale dimensions of an insulator in the conductivity calculations. Perhaps a much more convincing metric would be a comparison of actual power generation given similar dimensions and thermal

gradients, between 2D SSE, 3D SSE, and conventional thermoelectrics.

2. I am also concerned with the treatment of the 3D composite material as a thermally isotropic system, whereas the metallic nanostructure makes it highly anisotropic in thermal conductivity. Due to the thermal conductivity mismatch between the metallic layers and the insulating layers, would it not be expected that some of the thermal gradient be dropped across the metallic regions and therefore generate no SSE? For example, if a magnetic thin film was used to coat a powdered non-magnetic insulator, would we expect to see a large ANE signal still? Would a combination of both effects lead to compounding gains?

3. Some discussion is needed to compare the different benefits a 3D spin thermoelectric material may have over conventional 2D SSE geometries. Even if authors do not scale the devices for production, it would be useful to understand what new geometries and form factors are available that were not available before. A simple calculation based on scaling arguments may strengthen the claim that these materials may overtake either 2D SSE implementations or conventional thermoelectrics.

Version 2:

Reviewer comments:

Reviewer #2

(Remarks to the Author)
1) From Fig. 3, the E_y of YIG-Pt bulk composite in 10 mm size is about 100 µV/m under 5.8 K/mm, thus the transverse thermovoltage is about 1 µV. From the noise in Fig. 4, the transverse thermovoltage in the YIG-W bulk composite can be estimated as small as about 0.1 µV. The paper shows only two samples, and the revised paper does not include additional better experimental results. I worry about the reproducibility and stability of the spin Seebeck effect in bulk composites.
2) The spin Seebeck coefficient of YIG-Pt bulk composite is only 14 nV/K, 30 times smaller than 428 nV/K in conventional YIG/Pt. The bulk composite does not have better performance the conversional spin Seebeck device. What is internal resistance of bulk composite ? The power of bulk composite is as small as pW and could be over-estimated.

This work does not have good thermoelectric performance of device and new mechanism, thus I cannot recommend the publication of Nature Communications.

Reviewer #3

(Remarks to the Author)
The authors have fully addressed my concerns and I believe the manuscript is ready for publication at this point. I appreciated the additional of the scaling analysis, as it really enhances the clarity of presentation and highlights why this result is as transformative as it is.

Reviewer #4

(Remarks to the Author)
This work proposes and demonstrates a trans-scale spin Seebeck effect (SSE) in 3D YIG-Pt bulk composites, overcoming the thickness saturation limitation of conventional 2D thin-film SSE. The fabrication method, orthogonal magnetothermal configuration measurements, Pt/W contrast experiment, and power scaling analysis are well designed. I have read carefully the revised manuscript and the previous Reviewers' reports. I think the authors have better respond all the questions from Reviewers 2 and 3, and made responding revisions in the revised manuscript. I have only additional two questions before recommendation for publication in Nat Commun.
(1) The low thermal conductivity of the composite is attributed to interfacial phonon scattering, but lacks quantitative/qualitative decomposition from porosity, interface defects and grain boundary scattering. More detailed mechanism analysis is needed.
(2) Although XRD and magnetization measurements rule out major magnetic impurity phases, the possible magnetic proximity effect and local strain-induced magnetic order at YIG/Pt interface need further discussion.

Version 3:

Reviewer comments:

Reviewer #2

(Remarks to the Author)
I appreciate the authors' responses. It seems that the negative voltage was measured only in one YIG-W bulk composite. I reserve the opinions in my previous comments, but I will not prevent the publication.

Reviewer #4

(Remarks to the Author)
The authors have responded all my questions and made complete revisions, and I think the present version is acceptable for publication in Nat. Commun.

Open Access This Peer Review File is licensed under a Creative Commons Attribution 4.0 International License, which permits use, sharing, adaptation, distribution and reproduction in any medium or format, as long as you give appropriate credit to the original author(s) and the source, provide a link to the Creative Commons license, and indicate if changes were made.
In cases where reviewers are anonymous, credit should be given to 'Anonymous Referee' and the source.
The images or other third party material in this Peer Review File are included in the article's Creative Commons license, unless indicated otherwise in a credit line to the material. If material is not included in the article's Creative Commons license and your intended use is not permitted by statutory regulation or exceeds the permitted use, you will need to obtain permission directly from the copyright holder.
To view a copy of this license, visit https://creativecommons.org/licenses/by/4.0/

[Reply to the editor's and reviewers' comments]

We sincerely thank the handling editor and the reviewers for taking the time to review our manuscript and for providing thoughtful and constructive feedback. Below, we provide point-by-point responses to each of the reviewers' comments, along with the corresponding modifications made to the manuscript.

[Reply to the comments of Reviewer #1]

(Comment 1)

The manuscript reports a bulk, nanostructured Pt/YIG composite that exhibits isotropic, volumetric spin Seebeck effect (SSE). The authors fabricated conformal Pt shells on YIG powders by dynamic powder sputtering and then densified the powders by low-temperature, high-pressure hot pressing to create a three-dimensional network of Pt channels embedded in an insulating magnetic matrix. This architecture overcomes the thin-film constraint imposed by short electronic spin-diffusion length and finite magnon diffusion length, enabling SSE signals in two orthogonal magnetothermal geometries. The composite simultaneously attains much higher effective electrical conductivity than conventional YIG/Pt thin films while maintaining low thermal conductivity. Parasitic thermoelectric contributions are carefully minimized by field-odd symmetrization, structural/magnetic phase checks, and linear Hall responses.

The study addresses a central bottleneck of SSE devices, namely, thin-film geometry and interfacial area limitation, by scaling the conversion process from "interfacial" to "volumetric" in a mechanically robust bulk composite. The device concept, materials processing, and measurements are all convincing. This work clearly merits publication in Nature Communications. I therefore recommend acceptance after a single, focused minor revision that will substantially improve mechanistic clarity for readers.

(Response 1)

We sincerely thank the reviewer for the very careful reading of our manuscript and for the highly encouraging overall assessment. We are grateful for the constructive suggestions that helped us significantly improve the clarity of the mechanistic interpretation in our work. We respond to the reviewer's specific point below.

(Comment 1-1)

It would greatly benefit readers if the manuscript explicitly stated the symmetry-based reasoning that explains why local interfacial contributions add constructively, even in a complex Pt/YIG morphology, and why the macroscopic SSE remains aligned with $\nabla T \times \mathbf{m}$. My understanding is as follows:

1. For a local interfacial patch with unit normal $\hat{n}$ (pointing from YIG to Pt), the heat-driven spin injection across the interface scales with the normal component of the temperature gradient:

$$j_s^{int}(\hat{n}) \propto \hat{n} \cdot \nabla T$$

2. Inside Pt, the inverse spin Hall effect (ISHE) converts this spin current (flow direction $\parallel \hat{n}$) with spin polarization $\hat{m}$ into a local electric field:

$$E_{\mathrm{ISHE}} \propto \theta_{SH}(\hat{n} \times \hat{m})(\hat{n} \cdot \nabla T).$$

3. Crucially, under the inversion of the local normal $\hat{n} \to -\hat{n}$, both factors flip sign, and thus, their product does not. Hence, inner/outer facets of a Pt shell and rough/concave portions of the interface contribute with the same sign; they do not cancel.

4. If interface orientations are statistically isotropic, the orientational average leads to a simple

geometric factor (second-moment identity $\langle n_i n_j \rangle = \frac{\delta_{ij}}{3}$: $\langle (\hat{n} \times \hat{m})(\hat{n} \cdot \nabla T) \rangle = \frac{1}{3}(\nabla T \times \hat{m})$.
This shows that the macroscopic electric field is aligned with $(\nabla T \times \hat{m})$ and is only reduced by a scalar factor of 1/3, explaining why the volumetric contributions are additive even throughout a random network.

If my understanding above is inaccurate in any respect, I would ask the authors to correct it and briefly state the precise mechanism. Otherwise, I suggest that the authors include a short explanation of this reasoning, either in the main text near Fig. 1 or in the supplementary information. This would neatly bridge the conceptual gap from Fig. 1c to Fig. 1d.

(Response 1-1)

We sincerely thank the reviewer for this insightful and constructive suggestion. We fully agree that explicitly presenting the symmetry-based mechanism governing the constructive addition of local inverse spin Hall effect (ISHE) fields will help readers understand how a macroscopic SSE signal emerges from a geometrically disordered Pt/YIG interface network.

As the reviewer correctly summarized, the local ISHE field generated at an interface patch with unit normal $\hat{n}$ is proportional to

$$E_{\text{ISHE}}(\hat{n}) \propto (\hat{n} \cdot \nabla T)(\hat{n} \times \hat{m}), \qquad \text{(R1)}$$

so that both prefactors reverse sign simultaneously under inversion of the normal ($\hat{n} \to -\hat{n}$). Their product therefore remains invariant:

$$E_{\text{ISHE}}(\hat{n}) = E_{\text{ISHE}}(-\hat{n}), \qquad \text{(R2)}$$

which explains why inner versus outer surfaces, convex versus concave regions, and rough interface facets all contribute constructively rather than canceling.

To clarify the macroscopic response, we now explicitly include in the revised manuscript the orientational average for an isotropic distribution of interface normals. Under the assumption that $\hat{n}$ is statistically isotropic in the polycrystalline Pt/YIG composite, and using the identity $\langle n_i n_j \rangle = \frac{\delta_{ij}}{3}$, we obtain

$$\langle (\hat{n} \cdot \nabla T)(\hat{n} \times \hat{m}) \rangle = \frac{1}{3}(\nabla T \times \hat{m}), \qquad \text{(R3)}$$

showing that the emergent electric field is aligned with $(\nabla T \times \hat{m})$ and reduced only by a scalar geometric factor of 1/3.

A concise explanation of this symmetry argument has been added to the main text, and a more detailed derivation has been included in **Supplementary Note 1**, following the reviewer's helpful recommendation.

- On page 4, line 18 to page 5, line 14

"To elucidate the physical basis that enables the trans-scale SSE to survive such a geometrically complex FM/NM interface network, we first recall that in an FM/NM bilayer the ISHE field obeys

$$\mathbf{E}_{\text{ISHE}} \propto \mathbf{j}_s \times \hat{m} \qquad (1)$$

where $\mathbf{j}_s$ is the spatial direction of the thermally driven spin current induced by a temperature gradient ∇T, and $\hat{m}$ is the magnetization (spin-polarization) direction. For an arbitrarily oriented interface patch with unit normal $\hat{n}$, the injected spin current flows along $\hat{n}$, and its magnitude scales with the normal projection of ∇T. The local ISHE response therefore generalizes to

$$\mathbf{E}_{\mathrm{ISHE}}(\hat{n}) \propto (\hat{n}\cdot\nabla T)(\hat{n}\times\hat{m}). \tag{2}$$

Because both prefactors in **Equation. (2)** reverse sign under inversion of the interface normal ($\hat{n}\to-\hat{n}$), their product remains invariant, ensuring that rough, concave, convex, or oppositely oriented facets contribute constructively rather than cancelling. When the orientations of $\hat{n}$ are statistically isotropic in three-dimensional space, the orientational average reduces to

$$\langle(\hat{n}\cdot\nabla T)(\hat{n}\times\hat{m})\rangle = \frac{1}{3}(\nabla T\times\hat{m}) \tag{3}$$

indicating that the emergent macroscopic SSE retains the usual ISHE symmetry $\nabla T\times\hat{m}$ independent of morphological complexity, with a geometric reduction factor of 1/3. A full derivation is provided in **Supplementary Note 1**."

- On page 3 in Supplementary Information, we added **Supplementary Note 1**

"**Note S1. Symmetry-based derivation of macroscopic ISHE field**

Here we provide a brief derivation of **Equations (2)–(3)** in the main text, showing how a finite macroscopic inverse spin Hall effect (ISHE) field arises in a ferromagnetic material (FM)/normal metal (NM) composite with randomly oriented interface facets.

Local ISHE response for an arbitrary interface

For a local FM/NM interface patch with unit normal $\hat{n}$, the spin Seebeck effect (SSE) pumps a spin current perpendicular to the interface. Its local density can be expressed as

$$\mathbf{j}_s = j_0(\hat{n}\cdot\nabla T)\hat{n}, \tag{S1}$$

where ∇T is the applied temperature gradient and $(\hat{n}\cdot\nabla T)$ selects its normal component. The ISHE converts this spin current into a transverse electric field,

$$\mathbf{E}_{\mathrm{ISHE}}(\hat{n}) \propto \mathbf{j}_s(\hat{n})\times\hat{m} = (\hat{n}\cdot\nabla T)(\hat{n}\times\hat{m}), \tag{S2}$$

with the magnetization direction $\hat{m}$. Equation (S2) reproduces Eq. (2) in the main text. Under inversion of the interface normal ($\hat{n}\to\ -\hat{n}$), both factors in Eq. (S2) change sign,

$$(\hat{n}\cdot\nabla T)\to-(\hat{n}\cdot\nabla T),\qquad (\hat{n}\times\hat{m})\to-(\hat{n}\times\hat{m}), \tag{S4}$$

so their product, and thus the local ISHE field, remains unchanged:

$$\mathbf{E}_{\mathrm{ISHE}}(-\hat{n}) = \mathbf{E}_{\mathrm{ISHE}}(\hat{n}). \tag{S5}$$

This invariance explains why concave, convex, rough, or oppositely oriented interface facets contribute with the same sign.

Orientational average for an isotropic interface network

In a polycrystalline bulk composite, the FM/NM interfaces are oriented in all directions. For such an isotropic distribution of interface normals, the average products of their components along two spatial directions (i.e., $n_i n_j$) must reflect this symmetry. As a result, the orientational average takes the

rotationally symmetric form

$$\langle n_i n_j \rangle = \frac{1}{3}\delta_{ij}, \tag{S6}$$

meaning that the components of $\hat{n}$ are equally sampled in all directions. Averaging the local response in **Equation (S2)** using **Equation (S6)** yields

$$\langle \mathbf{E}_{\mathrm{ISHE}} \rangle \propto \frac{1}{3}(\nabla T \times \hat{m}), \tag{S7}$$

which is **Equation (3)** in the main text. This result shows that even in a geometrically complex FM/NM composite, the macroscopic SSE preserves the usual ISHE symmetry $\nabla T \times \hat{m}$ while being reduced only by a geometric factor of 1/3 due to isotropic averaging."

Importantly, we note that the reduction factor of 1/3 in **Equation (R3)** is not a fundamental limitation but a consequence of the isotropic distribution of interface normals. This observation suggests that the macroscopic SSE can be further enhanced by engineering anisotropic interface-orientation (i.e., $\hat{n}$) distribution. For example, if the YIG/Pt shell grains acquire anisotropic shapes or partial orientational alignment, e.g., during magnetic-field-assisted sintering, the distribution of $\hat{n}$ becomes biased rather than isotropic. In such cases, the orientational tensor $\langle n_i n_j \rangle$ deviates from $\frac{\delta_{ij}}{3}$, and the geometric factor can approach values larger than 1/3, with an upper bound of 1 corresponding to the planar thin-film limit.

To provide comprehensive guidance for future studies, we have added this point at the end of Discussion section in the revised manuscript.

- On page 13, lines 15–23, we added:

"From the extrinsic (structural) perspective, our symmetry analysis in **Equation (3)** reveals that the trans-scale SSE amplitude in the present composites contains a geometric reduction factor of 1/3 arising from the statistically isotropic distribution of local FMI/NM interface normals. Structural engineering that induces anisotropic or partially aligned interface orientations, for example, via controlled grain-shape anisotropy, magnetic-field-assisted forming, or directional sintering, can bias the distribution of interface normals and increase the geometric contribution toward the planar-film limit (i.e., the reduction factor is 1). This identifies structural orientation control as an additional, previously unexplored degree of freedom for amplifying trans-scale SSE signals in 3D architectures."

- To improve clarity, the future perspectives in the Discussion section were organized into two subsections: intrinsic materials engineering (pre-existing content) and extrinsic structural engineering (added in this revision).
- The overall structure of the Discussion section was reorganized to improve logical flow.

We sincerely appreciate the reviewer's contribution once again to improving the clarity of the manuscript.

[Reply to the comments of Reviewer #2]

(Comment 2)

The concept of bulk spin Seebeck composites has been reported in Ref. 43 Nat Commun 7, 13714 (2016), and thus it is not very new. This paper shows transverse thermoelectric voltages in Pt-YIG bulk composites. However, the spin Seebeck effect cannot be concluded by the results only with Pt. This paper does not show significant advance in physics understanding and clear advantage in performance. I cannot recommend the publication in Nature Communications. Please find my comments below.

(Response 2)

We sincerely thank the reviewer for carefully evaluating our manuscript. While we fully respect the reviewer's concerns regarding novelty and significance, we respectfully disagree with the assessment that our study does not provide a substantial advance in physics understanding or a fundamental advantage beyond Ref. 43 [*Nat Commun* **7**, 13714 (2016)].

As the reviewer correctly pointed out, Ref. 43 indeed introduced an important conceptual foundation for bulk SSE composites. However, the specific material platform used in that work relied on ferromagnetic metals (FMM) such as Ni and MnBi, which inherently impose severe constrains on trans-scale SSE realization. These FMMs possess extremely short spin diffusion lengths on the order of a few nanometers (for Ni, 3.5 nm), meaning that magnon-mediated spin transport would therefore require near-nanoscale control of the FMM phases, which is not feasible in macroscopic composites. Moreover, FMM such as MnBi are highly susceptible to oxidation, disrupting magnon transport and complicate reproducible SSE detection, as evidenced in Supplementary Section 3 of [*Joule* **5**, 3057–3067 (2021)].

In addition, Ref. 43 employed a precursor-based deposition route. Such chemical processing inevitably introduces reaction by-products at the interface, suppressing spin-mixing conductance by more than an order of magnitude, as shown in Ref. 44 [*Appl Phys Lett* **112**, 242403 (2018)]. Since interfacial spin transparency is essential for spin-thermoelectric conversion. These chemistry-driven limitations render it intrinsically challenging to isolate or optimize the SSE in bulk-composite architectures. Together, these limitations reflect long-standing, field-wide challenges in implementing a scalable and reliable bulk SSE platform.

Our work addresses these long-standing challenges by introducing a fundamentally different materials architecture based on a precursor-free ferromagnetic-insulator (FMI) platform with inherently long magnon-propagation length, combined with a conformal three-dimensional Pt shell network formed by sputtering. This approach avoids oxidation-driven degradation, eliminates precursor-induced interfacial contamination, and establishes continuous and spin-transparent FM/NM interfaces throughout the bulk. Supported by extensive control experiments that suppress the contribution of the anomalous Nernst effect (ANE) and confirm phase purity, our design enables reliable identification of isotropic volumetric SSE in a bulk composite.

This architectural and materials advance goes beyond addressing the limitations of Ref. 43; it provides a practical and scalable realization of a bulk SSE composite built on FMI-based magnon transport and clean FM/NM interfaces, which was previously inaccessible. We believe this framework represents a meaningful conceptual and experimental advance aligned with the publication criteria of *Nature Communications*.

To explicitly clarify this distinction in the revised manuscript, we incorporated the following modification:

- On page 6, lines 12–14, we added:

"While the conceptual structure of bulk SSE composites was outlined nearly a decade ago[43], their practical realization has remained difficult due to the intrinsic constraints discussed above."

Moreover, in the revised manuscript we have incorporated two additional experimental results that further strengthen the identification of the SSE origin in our bulk composites. First, we show that the transverse thermoelectric **voltage is suppressed at high magnetic fields**, consistent with the characteristic field dependence of the SSE rather than ANE-driven mechanisms. Second, we provide new measurements on **YIG–W composite** samples, in which W has a negative spin Hall angle. The **sign reversal of the transverse voltage** relative to the YIG–Pt case constitutes a stringent and independent verification of ISHE-driven spin–charge conversion. Taken together, these two observations provide experimental evidence for trans-scale SSE in bulk composites that, to our knowledge, has not previously been demonstrated. Details and corresponding modifications in the manuscript are provided in **Response 2-1**.

Finally, we would like to note that the revised manuscript now provides a substantially strengthened physical interpretation of the macroscopic SSE response. Following the suggestion from Reviewer #1, we incorporated a symmetry-based analysis that explicitly demonstrates how local ISHE fields generated at arbitrarily oriented YIG/Pt interfaces add constructively throughout the three-dimensional composite, and why the emergent electric field retains the characteristic $\nabla T \times \hat{m}$ symmetry with a geometric reduction factor of 1/3. This clarification resolves the conceptual ambiguity present in the earlier version and reinforces the mechanistic foundation of the trans-scale SSE demonstrated in this work.

A concise explanation of this symmetry argument has been added to the main text and a more detailed derivation has been included in **Supplementary Note S1**, following the reviewer's helpful recommendation.

- On page 4, line 18 to page 5, line 14

"To elucidate the physical basis that enables the trans-scale SSE to survive such a geometrically complex FM/NM interface network, we first recall that in an FM/NM bilayer the ISHE field obeys

$$\mathbf{E}_{\mathrm{ISHE}} \propto \mathbf{j}_s \times \hat{m} \qquad (1)$$

where $\mathbf{j}_s$ is the spatial direction of the thermally driven spin current induced by a temperature gradient ∇T, and $\hat{m}$ is the magnetization (spin-polarization) direction. For an arbitrarily oriented interface patch with unit normal $\hat{n}$, the injected spin current flows along $\hat{n}$, and its magnitude scales with the normal projection of ∇T. The local ISHE response therefore generalizes to

$$\mathbf{E}_{\mathrm{ISHE}}(\hat{n}) \propto (\hat{n} \cdot \nabla T)(\hat{n} \times \hat{m}). \qquad (2)$$

Because both prefactors in **Equation. (2)** reverse sign under inversion of the interface normal ($\hat{n} \rightarrow -\hat{n}$), their product remains invariant, ensuring that rough, concave, convex, or oppositely oriented facets contribute constructively rather than cancelling. When the orientations of $\hat{n}$ are statistically isotropic in three-dimensional space, the orientational average reduces to

$$\langle (\hat{n} \cdot \nabla T)(\hat{n} \times \hat{m}) \rangle = \frac{1}{3}(\nabla T \times \hat{m}) \qquad (3)$$

indicating that the emergent macroscopic SSE retains the usual ISHE symmetry $\nabla T \times \hat{m}$ independent of morphological complexity, with a geometric reduction factor of 1/3. A full derivation is provided in **Supplementary Note 1**."

- On page 3 in Supplementary Information, we added **Supplementary Note 1**

“**Note 1. Symmetry-based derivation of macroscopic ISHE field**

Here we provide a brief derivation of **Equations (2)–(3)** in the main text, showing how a finite macroscopic inverse spin Hall effect (ISHE) field arises in a ferromagnetic material (FM)/normal metal (NM) composite with randomly oriented interface facets.

Local ISHE response for an arbitrary interface

For a local FM/NM interface patch with unit normal $\hat{n}$, the spin Seebeck effect (SSE) pumps a spin current perpendicular to the interface. Its local density can be expressed as

$$\mathbf{j}_s = j_0(\hat{n} \cdot \nabla T)\hat{n}, \tag{S1}$$

where ∇T is the applied temperature gradient and $(\hat{n} \cdot \nabla T)$ selects its normal component. The ISHE converts this spin current into a transverse electric field,

$$\mathbf{E}_{\mathrm{ISHE}}(\hat{n}) \propto \mathbf{j}_s(\hat{n}) \times \hat{m} = (\hat{n} \cdot \nabla T)(\hat{n} \times \hat{m}), \tag{S2}$$

with the magnetization direction $\hat{m}$. Equation (S2) reproduces Eq. (2) in the main text. Under inversion of the interface normal ($\hat{n} \rightarrow -\hat{n}$), both factors in Eq. (S2) change sign,

$$(\hat{n} \cdot \nabla T) \rightarrow -(\hat{n} \cdot \nabla T), \qquad (\hat{n} \times \hat{m}) \rightarrow -(\hat{n} \times \hat{m}), \tag{S4}$$

so their product, and thus the local ISHE field, remains unchanged:

$$\mathbf{E}_{\mathrm{ISHE}}(-\hat{n}) = \mathbf{E}_{\mathrm{ISHE}}(\hat{n}). \tag{S5}$$

This invariance explains why concave, convex, rough, or oppositely oriented interface facets contribute with the same sign.

Orientational average for an isotropic interface network

In a polycrystalline bulk composite, the FM/NM interfaces are oriented in all directions. For such an isotropic distribution of interface normals, the average products of their components along two spatial directions (i.e., $n_i n_j$) must reflect this symmetry. As a result, the orientational average takes the rotationally symmetric form

$$\langle n_i n_j \rangle = \frac{1}{3}\delta_{ij}, \tag{S6}$$

meaning that the components of $\hat{n}$ are equally sampled in all directions. Averaging the local response in **Equation (S2)** using **Equation (S6)** yields

$$\langle \mathbf{E}_{\mathrm{ISHE}} \rangle \propto \frac{1}{3}(\nabla T \times \hat{m}), \tag{S7}$$

which is **Equation (3)** in the main text. This result shows that even in a geometrically complex FM/NM composite, the macroscopic SSE preserves the usual ISHE symmetry $\nabla T \times \hat{m}$ while being reduced only by a geometric factor of 1/3 due to isotropic averaging.”

The reviewer’s concern regarding the use of Pt is addressed in Response 2-1. Further point-by-point responses are provided below.

(Comment 2-1)

This paper shows transverse thermoelectric voltages only in Pt-YIG bulk composites in which the spin Hall angle of Pt is positive. The transverse thermoelectric voltages could be anomalous Nernst effect in

small amount of conductive Pt-doped YIG which is not detectable by XRD. To conclude the spin Seebeck and inverse spin Hall effects, the results should be also shown in the YIG bulk composites with nonmagnetic metal of negative spin Hall angle such as Ta and W.

(Response 2-1)

We sincerely thank the reviewer for raising this important point. We agree that, in principle, a transverse thermoelectric voltage measured in a Pt-containing composite could contain contributions from ANE if a conductive and magnetically active secondary phase were unintentionally formed. Although our structural analyses (XRD and TEM), magnetic measurements (VSM), and transport properties confirming the observation of the pure ordinary Hall effect without the anomalous Hall effect in our composite confirm no "effective" formation of any such phase, we appreciate the reviewer's suggestion that a comparison using a NM with a negative spin Hall angle would provide an unambiguous and complementary confirmation of the SSE origin.

In addition, we emphasize that the transverse thermoelectric voltage in the YIG–Pt composite (30-RT sample) is suppressed at high magnetic fields (see **Fig. 3f** in the main text). This field-induced suppression is a hallmark of the SSE, arising from the reduction of thermally excited magnons, and is not consistent with ANE-driven mechanisms, which do not exhibit comparable high-field damping. This behavior provides an independent, physics-based signature supporting the SSE origin of our signals.

Motivated by this recommendation, we prepared an additional set of YIG-based bulk composites coated with W, which has a negative spin Hall angle. The transverse thermoelectric response of this W–YIG composite exhibits a clear sign reversal relative to the Pt–YIG composite. This sign inversion directly follows the expected ISHE symmetry and is inconsistent with any ANE scenario.

These new results, (1) high-field suppression of the voltage, and (2) sign reversal in the YIG–W composite, provide strong and direct evidence that the observed transverse voltages originate from the magnon-induced spin pumping and its conversion via ISHE. We have incorporated both data sets into the revised manuscript, as detailed below.

(1) High-field data

- On page 17, **Figure 3f** (previously showing magnetization data) has been replaced with newly added high-field SSE data of the 30-RT sample.

“

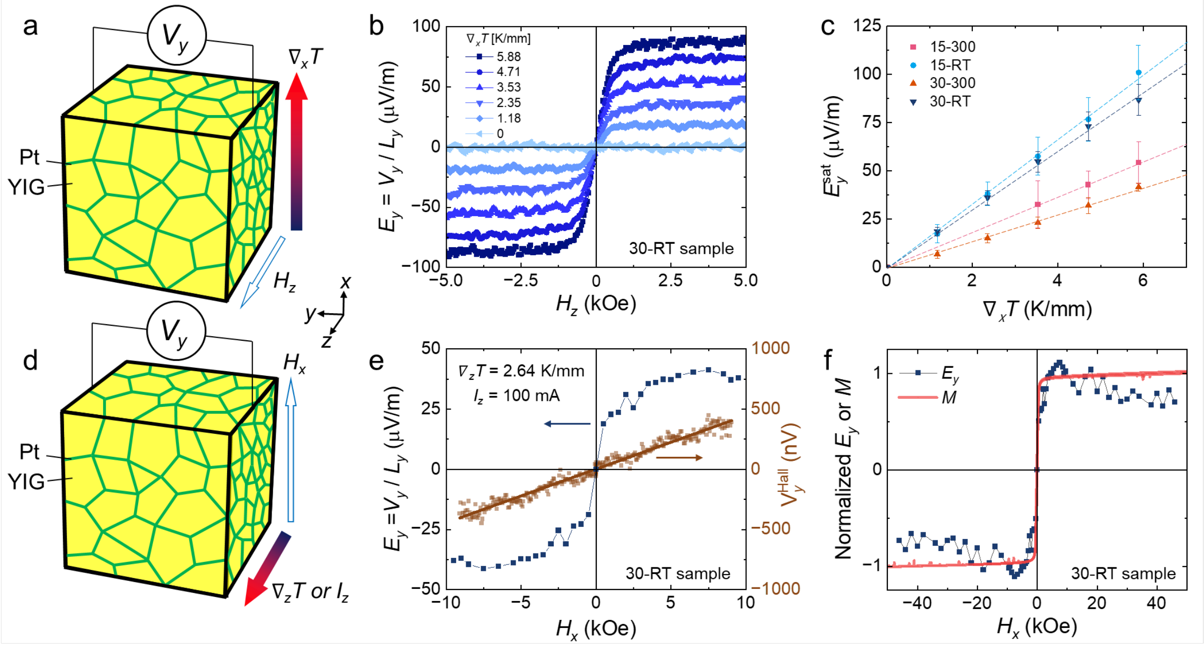

Fig. 3| Observation of the trans-scale spin Seebeck effect. (a) Schematic illustrations of H_z-$\nabla_x T$

configuration. (b) Measured transverse electric field (E_y) under ∇T in H_z-$\nabla_x T$ configuration as a function of H. The subscripts x, y, and z indicate the corresponding directions of the applied temperature gradient (∇T), charge current (I), and magnetic field (H). (c) Saturated electric field (E_y^{Sat}) plotted as a function of $\nabla_x T$. (d) Schematic illustrations of H_x-$\nabla_z T$ configuration. (e) Measured transverse electric field (E_y) under ∇T in H_x-$\nabla_z T$ configuration. The measured Hall voltage (V_y^{Hall}) is also presented for reference, along with a linear fitting curve. (f) Normalized E_y and Magnetization (M) of the 30-RT sample under high H. Detailed M-H curves for the other samples are provided in **Supplementary Fig. 6**."

- On page 11, line 21 to page 12, line 3 (Discussion), we added:
  "Furthermore, the transverse thermoelectric signal exhibits a suppression at high magnetic fields (**Fig. 3f**), which is a well-established hallmark of the SSE[13,15]. This suppression arises from the field-induced reduction of thermally excited magnons and is not expected for ANE of FMM impurities, whose transverse voltage simply follows the magnetization and saturates at high fields. This high-field damping therefore provides an additional signature that the observed transverse voltage originates from magnon-driven SSE rather than ANE."
- The previous **Fig. 3f** (magnetization data) has been moved to **Supplementary Fig. 6a** (page 13 of the Supplementary Information).

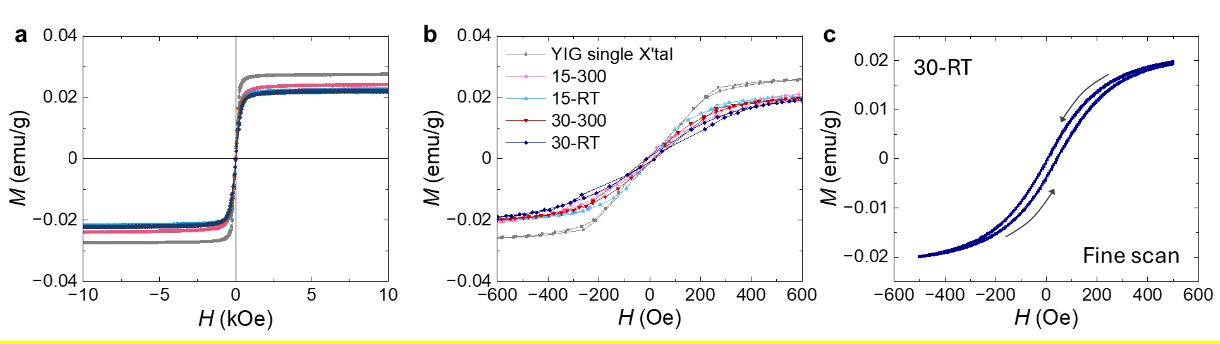

Supplementary Fig. 6| Magnetization curves of the samples. (a) High-field and (b) low-field magnetization data of the samples. (c) High-resolution magnetization curve of the 30-RT sample obtained with find field steps within the low field range.

(2) YIG–W data

- On page 12, lines 4–10 (Discussion), we added:

"Finally, to provide an independent and unambiguous confirmation, we prepared a YIG–W composite, where W has a negative spin Hall angle. **Figure 4** shows that the transverse thermoelectric voltage of the YIG–W composite in the H_z-$\nabla_x T$ configuration exhibits a clear sign reversal relative to the YIG–Pt composite under otherwise identical conditions. This inversion provides direct evidence that the observed transverse voltage originates from the magnon-driven thermal spin pumping and its ISHE-driven spin-charge conversion in the bulk composite, establishing the trans-scale SSE."

- To improve the logical flow, we also reorganized the order of paragraphs within the Discussion section.
- In the main text (page 18), we added **Fig. 4**:

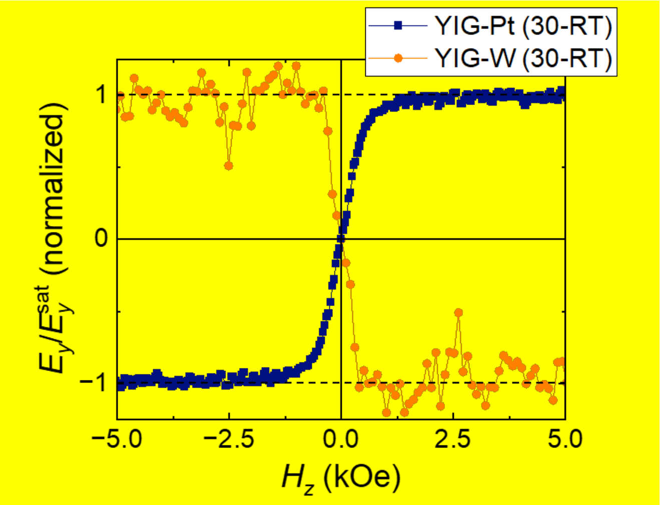

Fig. 4| Normalized spin Seebeck voltage for YIG–Pt and YIG–W bulk composites. The two samples exhibit opposite signs of transverse thermoelectric voltage, reflecting the opposite signs of the spin Hall angles of the nonmagnetic metals, Pt (positive) and W (negative). The signal in the YIG–W composite appears noisier due to the large electrical resistance of the non-optimized composite. See Supplementary Note S5 for fabrication details and discussion.

- The experimental details are included in Supplementary Note 5, as follows:

"Note 5. Observation of trans-scale SSE in YIG–W bulk composite

To unambiguously verify the origin of the transverse thermoelectric voltage observed in the YIG–Pt composites, we performed a control experiment by replacing Pt with W, which has a negative spin Hall angle. The YIG–W composite was fabricated using the same procedure as the YIG–Pt samples (specifically the 30-RT condition). W was deposited onto the surface of YIG powders using the powder sputtering system and subsequently consolidated by high-pressure pressing at 500 MPa, identical to the procedure used for the Pt-based composite. The resulting YIG–W composite exhibited higher mechanical brittleness than the YIG–Pt samples, possibly due to the non-optimized fabrication conditions and/or partial oxidation of the W shells. The electrical resistance of the sample exceeded 10 kΩ, more than four orders of magnitude higher than that of the YIG–Pt composite with similar dimensions, suggesting that the W network was not fully continuous throughout the bulk. Further optimization may be required to obtain mechanically robust, electrically connected YIG–W composites. Due to the mechanical brittleness of the YIG–W composite, the samples could not be cut into well-defined rectangular geometries. Measurements were therefore performed using irregular pellet-like pieces, which makes absolute normalization by geometric dimensions challenging. To allow a meaningful comparison with the YIG–Pt results, the data presented in **Fig. 4** of the main text are shown in a normalized for. Despite these non-idealities, we clearly observed a transverse thermoelectric voltage with the opposite sign compared with the YIG–Pt composite, as shown in **Fig. 4** in the main text. Although the signal is noisier possibly due to the high resistance of the sample, the sign reversal is evident and exceeds the fluctuation level. This behavior is fully consistent with the expected sign change of the spin Hall angle when replacing Pt (positive) with W (negative), thereby providing direct and independent evidence that the transverse thermoelectric voltage originates from magnon-driven thermal spin pumping in YIG and the spin-charge conversion via the ISHE in the NM."

We appreciate the reviewer for prompting this constructive addition, which has further strengthened the manuscript.

(Comment 2-2)

The spin Seebeck coefficient is 17 nV/K in the Pt-YIG bulk composites, which is 30 times smaller than that of the conventional Pt/YIG layered structure (about 0.5 µV/K). The bulk composites do not show advantage in the thermoelectric coefficient.

(Response 2-2)

We thank the reviewer for this comment. While the reported spin Seebeck coefficient (SSC) in the present Pt/YIG bulk composite (17 nV/K) is indeed smaller than that of optimized thin-film Pt/YIG bilayers, we respectfully point out that the thermoelectric coefficient alone does not capture the functional significance or performance potential of trans-scale SSE architectures.

First, thin-film Pt/YIG bilayers inherently operate in a quasi-two-dimensional geometry where only a narrow interfacial region contributes to the SSE-based thermoelectric conversion. Their SSC is evaluated per unit interfacial area and does not scale with device volume. In contrast, bulk composites generate and convert spin currents throughout a three-dimensional FMI/NM network, enabling volumetric energy conversion and macroscopic electrical conduction pathways, capabilities fundamentally inaccessible to thin-film structures. Therefore, a direct comparison of the coefficient magnitude does not reflect the architectural advantages or intended application space of the trans-scale SSE platform.

Second, the present study does not yet represent an optimized bulk SSE system. The Pt-shell thickness, shell continuity, densification conditions, grain microstructure, and FMI/NM connectivity were selected to establish a precursor-free, structurally continuous bulk architecture, not to maximize the SSC. As widely observed in spin-caloritronic thin films, meaningful enhancement typically requires substantial iterative materials and interface optimization. We expect that systematic tuning of these parameters will significantly improve the SSC of the bulk composite, as has been historically achieved in thin-film SSE systems.

Third, as now clarified in the Discussion of the revised manuscript, the present composite exhibits a *geometric reduction factor* of 1/3 that arises from the statistically isotropic distribution of local FMI/NM interface normals. This reduction is not an intrinsic limitation of the bulk architecture. Structural orientation engineering, such as inducing partial alignment of interface normals via anisotropic grain shaping, magnetic-field-assisted pressing, or directional sintering, could increase this geometric contribution toward the planar-film limit (factor of 1). This provides a clear and experimentally accessible route for future enhancement of the bulk SSC.

Finally, the conceptual advance of our work lies not in achieving the highest SSC at this initial stage but in establishing a scalable, precursor-free FMI-based bulk architecture that preserves clean interfaces, supports long-range magnon transport, and enables isotropic volumetric SSE. This architectural novelty and platform-level advance cannot be evaluated solely by comparison with fully optimized thin-film values.

To clarify these points, we have added explanatory sentences in the revised Discussion section addressing (i) the distinction between coefficient magnitude and volumetric operation, (ii) the current lack of performance optimization, and (iii) the geometric reduction factor and its potential mitigation through structural engineering.

- On page 13, line 22 to page 14, line7, we added:
  "From the extrinsic (structural) perspective, our symmetry analysis in **Equation (3)** reveals that the trans-scale SSE amplitude in the present composites contains a geometric reduction factor of 1/3 arising from the statistically isotropic distribution of local FMI/NM interface normals. Structural engineering that induces anisotropic or partially aligned interface orientations, for example, via controlled grain-shape anisotropy, magnetic-field-assisted forming, or directional sintering, can bias the distribution of interface normals and increase the geometric contribution toward the planar-film limit (i.e., the reduction factor is 1). This identifies structural orientation control as an additional, previously unexplored degree of freedom for amplifying trans-scale SSE signals in 3D architectures."
- To improve clarity, the future perspectives in the Discussion section were organized into two

subsections: intrinsic materials engineering (pre-existing content) and extrinsic structural engineering (added in this revision).

- The overall structure of the Discussion section was reorganized to improve logical flow.

We appreciate the reviewer for raising this point.

(Comment 2-3)

In Table S1, what differences are the devices with the four effective electrical conductivities (σeff) for this work ? It is not meaningful to show the "effective" electrical conductivity of Pt/YIG by normalizing with the large geometric dimensions of insulating YIG. In fact, the electrical conductivity of the bulk composite is as poor as 10^2 S/m, which is 4 orders smaller than the electrical conductivity of the Pt layer 10^6 S/m.

(Response 2-3)

We thank the reviewer for raising this question. We respectfully clarify that the effective electrical conductivity (σ^{eff}) in Table S1 is introduced **to provide a consistent and device-level basis** for comparing electrical performance across different SSE platforms, including both thin-film FMI/NM structures and bulk composites.

In conventional SSE studies, the intrinsic conductivity of the Pt layer (~ 10^6 S/m) is often quoted, but this quantity does **not** reflect the electrical performance of an actual device. The output power is governed by the total device resistance, which depends on the full device thickness including the typically millimeter-scale FM and substrate in thin-film structures. When the conductance of thin-film devices is normalized by the actual device dimensions, their σ^{eff} values fall in the range of 2–60 S/m (**Table S1**), several orders of magnitude smaller than σ_{Pt}, despite having a high-quality Pt layer. This normalization is not an artifact of our composite geometry but reflects the actual electrical pathway that determines the usable power output of any SSE device.

For this reason, σ^{eff} is the appropriate metric for evaluating SSE devices: it reflects the effective macroscopic conductivity of the entire electrically active pathway, regardless of whether the device is a thin-film stack or a bulk composite. This allows direct comparison of electrical performance across architectures.

In our YIG-Pt composites, the formation of microscale percolating Pt pathways leads to $\sigma^{eff} = (5–50) \times 10^2$ S/m, which is 2–3 orders of magnitude higher than σ^{eff} of conventional thin-film FMI/NM devices calculated on the same device-normalized basis. Thus, σ^{eff} is meaningful not as an intrinsic material property but as a device-level quantity that captures the macroscopic conduction path relevant for output power and circuit modeling.

To prevent misunderstanding, we have added clarifications in **Supplementary Note S4** emphasizing that σ^{eff} is a device-level performance parameter and is intentionally used to compare thin-film and bulk SSE platforms on equal footing.

- On page 6 of Supplementary Information (Supplementary Note 4), we added:

"Here, σ^{eff} does not reflect the intrinsic conductivity of NM, but rather the device-level macroscopic conductivity determined by the geometry, volume fraction, and connectivity of the NM network within the composite."

[List of corrections]

Main text

Page 4, Line 18–Page 5, Line 14.
(Added sentences)
To elucidate the physical basis that enables the trans-scale SSE to survive such a geometrically complex FM/NM interface network, we first recall that in an FM/NM bilayer the ISHE field obeys

$$\mathbf{E}_{\mathrm{ISHE}} \propto \mathbf{j}_s \times \hat{m} \quad (1)$$

where $\mathbf{j}_s$ is the spatial direction of the thermally driven spin current induced by a temperature gradient ∇T, and $\hat{m}$ is the magnetization (spin-polarization) direction. For an arbitrarily oriented interface patch with unit normal $\hat{n}$, the injected spin current flows along $\hat{n}$, and its magnitude scales with the normal projection of ∇T. The local ISHE response therefore generalizes to

$$\mathbf{E}_{\mathrm{ISHE}}(\hat{n}) \propto (\hat{n} \cdot \nabla T)(\hat{n} \times \hat{m}). \quad (2)$$

Because both prefactors in **Equation. (2)** reverse sign under inversion of the interface normal ($\hat{n} \to -\hat{n}$), their product remains invariant, ensuring that rough, concave, convex, or oppositely oriented facets contribute constructively rather than cancelling. When the orientations of $\hat{n}$ are statistically isotropic in three-dimensional space, the orientational average reduces to

$$\langle (\hat{n} \cdot \nabla T)(\hat{n} \times \hat{m}) \rangle = \frac{1}{3}(\nabla T \times \hat{m}) \quad (3)$$

indicating that the emergent macroscopic SSE retains the usual ISHE symmetry $\nabla T \times \hat{m}$ independent of morphological complexity, with a geometric reduction factor of 1/3. A full derivation is provided in **Supplementary Note 1**.

Page 6, Lines 12–14.
(Added sentence)
While the conceptual structure of bulk SSE composites was outlined nearly a decade ago[43], their practical realization has remained difficult due to the intrinsic constraints discussed above.

Page 11, Line 21–Page 12, Line 10.
(Added sentences)
Furthermore, the transverse thermoelectric signal exhibits a suppression at high magnetic fields (**Fig. 3f**), which is a well-established hallmark of the SSE[13,15]. This suppression arises from the field-induced reduction of thermally excited magnons and is not expected for ANE of FMM impurities, whose transverse voltage simply follows the magnetization and saturates at high fields. This high-field damping therefore provides an additional signature that the observed transverse voltage originates from magnon-driven SSE rather than ANE.
Finally, to provide an independent and unambiguous confirmation, we prepared a YIG–W composite, where W has a negative spin Hall angle. **Figure 4** shows that the transverse thermoelectric voltage of the YIG–W composite in the H_z-$\nabla_x T$ configuration exhibits a clear sign reversal relative to the YIG–Pt composite under otherwise identical conditions. This inversion provides direct evidence that the observed transverse voltage originates from the magnon-driven thermal spin pumping and its ISHE-driven spin-charge conversion in the bulk composite, establishing the trans-scale SSE.

Page 13, Line 4–7.
(Original manuscript)
To further enhance device output power, future strategies may include optimizing FM/NM interfaces[21,22], for example by inserting topological materials[34,68] or organic semiconductors[69] at the interface, as well as engineering of magnon transport in YIG independently from phonons[16,21].

(Revised manuscript)
From the intrinsic (materials) perspective, further improvement in output power can be achieved by optimizing FM/NM interfaces[21,22], for example by inserting topological materials[34,68] or organic

semiconductors[69] at the interface, as well as engineering magnon transport in YIG independently from phonons[16,21].

Page 13, Line 22–Page 14, Line 15.

(Original manuscript)

Altogether, the demonstration of trans-scale SSE opens new avenues for transverse thermoelectrics based on spin currents.

(Revised manuscript)

From the extrinsic (structural) perspective, our symmetry analysis in **Equation (3)** reveals that the trans-scale SSE amplitude in the present composites contains a geometric reduction factor of 1/3 arising from the statistically isotropic distribution of local FMI/NM interface normals. Structural engineering that induces anisotropic or partially aligned interface orientations, for example, via controlled grain-shape anisotropy, magnetic-field-assisted forming, or directional sintering, can bias the distribution of interface normals and increase the geometric contribution toward the planar-film limit (i.e., the reduction factor is 1). This identifies structural orientation control as an additional, previously unexplored degree of freedom for amplifying trans-scale SSE signals in 3D architectures.

Altogether, the demonstration of trans-scale SSE in a bulk composite platform establishes a previously inaccessible regime of transverse thermoelectric conversion that extends well beyond the constraints of conventional thin-film architectures. By enabling volumetric spin-current generation and macroscopic ISHE conversion within a mechanically robust 3D network, this work provides a practical and scalable route for implementing spin-based thermoelectric functionality at bulk scale. These results position trans-scale SSE composites as a promising platform for next-generation transverse thermoelectrics based on spin currents.

- **Figure 3f has been revised.**

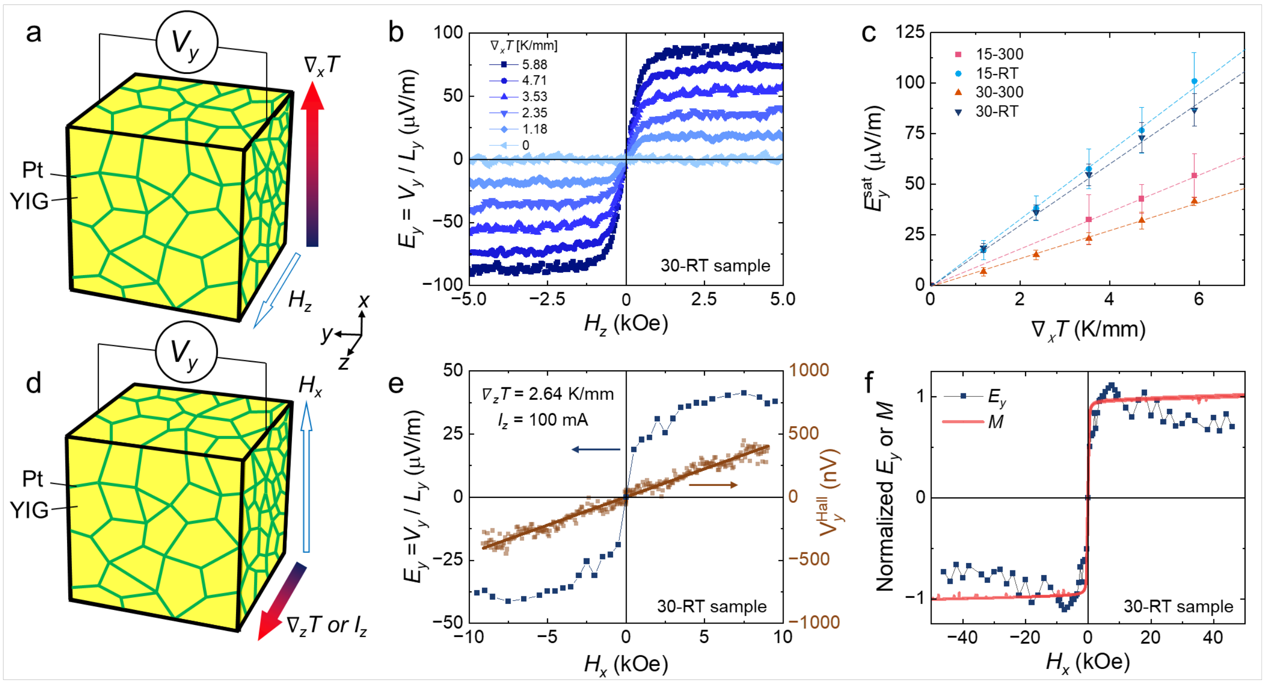

Fig. 3| Observation of the trans-scale spin Seebeck effect. (a) Schematic illustrations of H_z-$\nabla_x T$ configuration. (b) Measured transverse electric field (E_y) under ∇T in H_z-$\nabla_x T$ configuration as a function of H. The subscripts x, y, and z indicate the corresponding directions of the applied temperature gradient (∇T), charge current (I), and magnetic field (H). (c) Saturated electric field (E_y^{Sat}) plotted as a function of $\nabla_x T$. (d) Schematic illustrations of H_x-$\nabla_z T$ configuration. (e) Measured transverse electric field (E_y) under ∇T in H_x-$\nabla_z T$ configuration. The measured Hall voltage (V_y^{Hall}) is also presented for reference, along with a linear fitting curve. (f) Normalized E_y

and Magnetization (*M*) of the 30-RT sample under high *H*. Detailed *M-H* curves for the other samples are provided in **Supplementary Figure 6**.

- **Figure 4 has been added.**

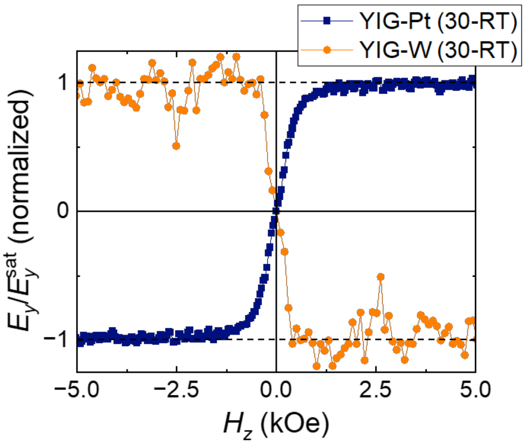

Fig. 4| Normalized spin Seebeck voltage for YIG–Pt and YIG–W bulk composites. The two samples exhibit opposite signs of transverse thermoelectric voltage, reflecting the opposite signs of the spin Hall angles of the nonmagnetic metals, Pt (positive) and W (negative). The signal in the YIG–W composite appears noisier due to the large electrical resistance of the non-optimized composite. See Supplementary Note 5 for fabrication details and discussion.

- **In accordance with the editorial request, we have thoroughly reformatted the manuscript.**
  - Specifically, all supplementary labels have been standardized by replacing Fig. SX, Note SX, and Table SX with the required forms Supplementary Fig. X, Supplementary Note X, and Supplementary Table X, respectively. These changes have been applied consistently throughout both the main text and the Supplementary Information.
  - Furthermore, typographical emphasis that was previously applied to terms such as *trans-scale SSE* has been removed, following the formatting guidelines.
  - Experimental section has been replaced with Methods.

- **We have newly added Supplementary Note 1.**

Note S1. Symmetry-based derivation of macroscopic ISHE field

Here we provide a brief derivation of **Equations (2)–(3)** in the main text, showing how a finite macroscopic inverse spin Hall effect (ISHE) field arises in a ferromagnetic material (FM)/normal metal (NM) composite with randomly oriented interface facets.

Local ISHE response for an arbitrary interface

For a local FM/NM interface patch with unit normal $\hat{n}$, the spin Seebeck effect (SSE) pumps a spin current perpendicular to the interface. Its local density can be expressed as

$$\mathbf{j}_s = j_0(\hat{n} \cdot \nabla T)\hat{n}, \tag{S1}$$

where ∇T is the applied temperature gradient and $(\hat{n} \cdot \nabla T)$ selects its normal component. The ISHE converts this spin current into a transverse electric field,

$$\mathbf{E}_{\mathrm{ISHE}}(\hat{n}) \propto \mathbf{j}_s(\hat{n}) \times \hat{m} = (\hat{n} \cdot \nabla T)(\hat{n} \times \hat{m}), \tag{S2}$$

with the magnetization direction $\hat{m}$. Equation (S2) reproduces Eq. (2) in the main text. Under inversion of the interface normal ($\hat{n} \rightarrow -\hat{n}$), both factors in Eq. (S2) change sign,

$$(\hat{n} \cdot \nabla T) \rightarrow -(\hat{n} \cdot \nabla T), \qquad (\hat{n} \times \hat{m}) \rightarrow -(\hat{n} \times \hat{m}), \tag{S4}$$

so their product, and thus the local ISHE field, remains unchanged:

$$\mathbf{E}_{\mathrm{ISHE}}(-\hat{n}) = \mathbf{E}_{\mathrm{ISHE}}(\hat{n}). \tag{S5}$$

This invariance explains why concave, convex, rough, or oppositely oriented interface facets contribute with the same sign.

Orientational average for an isotropic interface network

In a polycrystalline bulk composite, the FM/NM interfaces are oriented in all directions. For such an isotropic distribution of interface normals, the average products of their components along two spatial directions (i.e., $n_i n_j$) must reflect this symmetry. As a result, the orientational average takes the rotationally symmetric form

$$\langle n_i n_j \rangle = \frac{1}{3}\delta_{ij}, \tag{S6}$$

meaning that the components of $\hat{n}$ are equally sampled in all directions. Averaging the local response in **Equation (S2)** using **Equation (S6)** yields

$$\langle \mathbf{E}_{\mathrm{ISHE}} \rangle \propto \frac{1}{3}(\nabla T \times \hat{m}), \tag{S7}$$

which is **Equation (3)** in the main text. This result shows that even in a geometrically complex FM/NM composite, the macroscopic SSE preserves the usual ISHE symmetry $\nabla T \times \hat{m}$ while being reduced only by a geometric factor of 1/3 due to isotropic averaging.

Page 6 of Supplementary Information.

(Added sentences)

Here, σ^{eff} does not reflect the intrinsic conductivity of NM, but rather the device-level macroscopic conductivity determined by the geometry, volume fraction, and connectivity of the NM network within the composite.

- **We have newly added Supplementary Note 5.**

Note 5. Observation of trans-scale SSE in YIG–W bulk composite

To unambiguously verify the origin of the transverse thermoelectric voltage observed in the YIG–Pt composites, we performed a control experiment by replacing Pt with W, which has a negative spin Hall angle. The YIG–W composite was fabricated using the same procedure as the YIG–Pt samples (specifically the 30-RT condition). W was deposited onto the surface of YIG powders using the powder sputtering system and subsequently consolidated by high-pressure pressing at 500 MPa, identical to the procedure used for the Pt-based composite. The resulting YIG–W composite exhibited higher mechanical brittleness than the YIG–Pt samples, possibly due to the non-optimized fabrication conditions and/or partial oxidation of the W shells. The electrical resistance of the sample exceeded 10 kΩ, more than four orders of magnitude higher than that of the YIG–Pt composite with similar dimensions, suggesting that the W network was not fully continuous throughout the bulk. Further optimization may be required to obtain mechanically robust, electrically connected YIG–W composites. Due to the mechanical brittleness of the YIG–W composite, the samples could not be cut into well-defined rectangular geometries. Measurements were therefore performed using irregular pellet-like pieces, which makes absolute normalization by geometric dimensions challenging. To allow a meaningful comparison with the YIG–Pt results, the data presented in **Fig. 4** of the main text are shown in a normalized for. Despite these non-idealities, we clearly observed a transverse thermoelectric voltage with the opposite sign compared with the YIG–Pt composite, as shown in **Fig. 4** in the main text. Although the signal is noisier possibly due to the high resistance of the sample, the sign reversal is evident and exceeds the fluctuation level. This behavior is fully consistent with the expected sign change of the spin Hall angle when replacing Pt (positive) with W (negative), thereby providing direct and independent evidence that the transverse thermoelectric voltage originates from magnon-driven thermal spin pumping in YIG and the spin-charge conversion via the ISHE in the NM.

- **The previous Fig. 3f (magnetization data) has been moved to Supplementary Fig. 6a (page 13 of the Supplementary Information).**

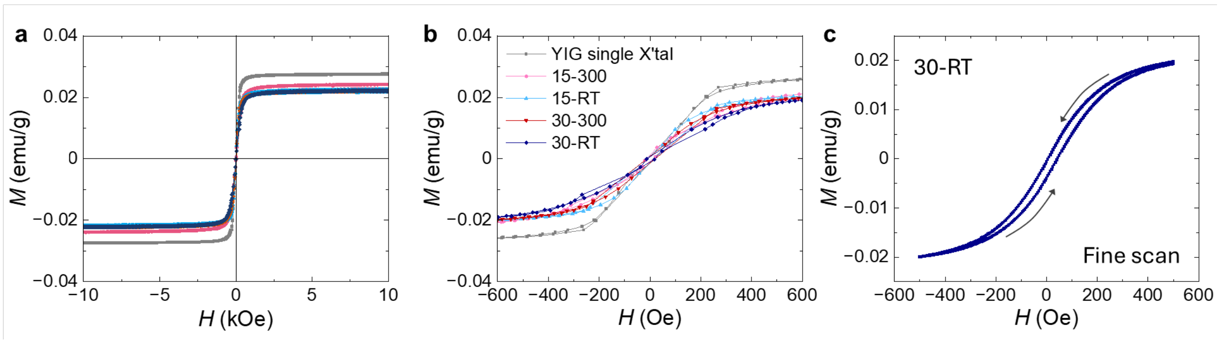

Supplementary Fig. 6| Magnetization curves of the samples. (a) High-field and (b) low-field magnetization data of the samples. (c) High-resolution magnetization curve of the 30-RT sample obtained with find field steps within the low field range.

[Reply to the editor's and reviewers' comments]

We sincerely thank the handling editor and the reviewers for taking the time to review our manuscript and for providing thoughtful and constructive feedback. Below, we provide point-by-point responses to each of the reviewers' comments, along with the corresponding modifications made to the manuscript.

[Reply to the comments of Reviewer #1]

(Comment 1)

I have reviewed the revised manuscript and the authors' response. The explicit inclusion of the symmetry-based reasoning for the constructive addition of local ISHE fields, together with the detailed derivation in the main text and Supplementary Note 1, has clarified the mechanism and fully addressed my concerns. I recommend this manuscript for publication in Nature Communications.

(Response 1)

We sincerely thank the Reviewer for the careful evaluation of our revised manuscript and for the positive assessment. We are pleased that the inclusion of the symmetry-based reasoning and the detailed derivation clarified the mechanism and addressed the prior concerns. We appreciate the Reviewer's recommendation for publication.

[Reply to the comments of Reviewer #2]

(Comment 2-1)

As the authors' response, the concept of bulk spin Seebeck composites is not new. The results are the spin Seebeck effect based on magnetic insulator which has been intensively studied. Again, this paper does not show significant advance in physics understanding.

(Response 2-1)

We thank the Reviewer for this comment. We agree that the SSE in magnetic insulators has been extensively studied in thin-film geometries, and that early demonstrations of bulk SSE composites were reported previously. We respectfully clarify, however, that the contribution of the present work is not the introduction of a new microscopic spin-caloritronic mechanism.

Rather, the advance demonstrated here concerns both the dimensional transport regime and scalability of spin thermoelectric conversion. Following the initial bulk-composite demonstrations reported in 2016, the field has remained dominated by interfacial thin-film SSE geometries, and a quantitative power-scaling framework for bulk SSE architectures has not been established. Moreover, reports demonstrating a percolated, volumetrically connected ISHE-active metallic network inside a ferrimagnetic-insulator bulk composite have not emerged. This gap has limited progress toward architecturally well-defined and scalable bulk SSE implementations.

Importantly, to the best of our knowledge, the present study provides the first experimental demonstration of bulk-scale spin Seebeck signals that are rigorously verified to originate from magnon-mediated SSE without parasitic contributions. Through high-field suppression behavior, sign reversal using a negative spin Hall angle metal, and systematic exclusion of parasitic transverse thermoelectric effects, we confirm that the observed transverse voltage arises from magnon-mediated spin-to-charge

conversion alone. While prior bulk-type systems reported transverse thermoelectric responses, unambiguous experimental verification of a pure SSE signal at the millimeter-scale bulk level has remained limited.

In contrast, the present work experimentally establishes and validates a geometry-controlled volumetric scaling regime for spin thermoelectric conversion. We therefore respectfully submit that this study represents an important advance over prior bulk SSE implementations by addressing a persistent architectural bottleneck that has limited the transition from interfacial thin films to scalable bulk-compatible systems.

(Comment 2-2)

The signal in Fig. 3f is noisy at high field for the YIG-Pt bulk composite. The signal of YIG-W bulk composite in Fig. 4 is also noisy. Why not show Ey for these results ? The performance of bulk spin Seebeck composite is really poor in this work.

(Response 2-2)

We thank the Reviewer for this comment.

First, regarding **Fig. 3f**, the physically relevant feature of the high-field data is the systematic decrease of the transverse voltage with increasing magnetic field. This monotonic suppression reflects the reduction of the SSE due to magnon gap opening. The decreasing trend is the key observation and is fully consistent with the expected field dependence of SSE.

Second, for **Fig. 4**, the central result is the clear polarity reversal observed in the YIG–W composite compared to YIG–Pt. This sign change directly supports a SSE-driven inverse spin Hall origin of the transverse voltage rather than the ANE. Although the YIG–W samples exhibit reduced signal-to-noise ratio, due to the higher internal resistance exceeding 10 kΩ compared to the YIG-Pt composite, the polarity reversal and magnetic-field symmetry remain clearly resolved. Detailed experimental considerations, including the higher resistance of the YIG–W samples and sample preparation aspects, are described in **Supplementary Note 4**.

Third, we emphasize that the measurements shown in **Fig. 3f** and **Fig. 4** were conducted in direct response to the Reviewer's previous Comment 2-1 "This paper shows transverse thermoelectric voltages only in Pt-YIG bulk composites in which the spin Hall angle of Pt is positive. The transverse thermoelectric voltages could be anomalous Nernst effect in small amount of conductive Pt-doped YIG which is not detectable by XRD. To conclude the spin Seebeck and inverse spin Hall effects, the results should be also shown in the YIG bulk composites with nonmagnetic metal of negative spin Hall angle such as Ta and W," requesting verification using a nonmagnetic metal with a negative spin Hall angle. These experiments were designed to clarify the physical origin of the observed signal, rather than to provide optimized performance metrics of bulk SSE devices.

Finally, the absolute E_y value was not presented for the YIG–W data (**Fig. 4**) because the samples were mechanically brittle and measured in pellet form. Under these conditions, reliable cutting into well-defined rectangular geometries was not feasible, preventing accurate geometric normalization required for precise E_y extraction. Therefore, the data were presented in terms of normalized saturation values, which is described in **Supplementary Note S4**.

(Comment 2-3)

I cannot find the authors' response on the differences in the devices with the four effective electrical conductivities (σeff) from 5 x 10^2 S/m to 49 x 10^2 S/m for this work in Table S1. It is strange to compare the "effective" electrical conductivity of Pt/YIG by normalizing with the large geometric dimensions of insulating YIG.

(Response 2-3)

We thank the Reviewer for this comment.

The differences in the four previously reported effective electrical conductivities (5–49 × 10^2 S/m) arise primarily from variations in Pt volume fraction and interparticle connectivity within the composite network depending on different sintering conditions. In these bulk composites, electrical transport is governed by percolation through nanoscale Pt coatings surrounding YIG. Small variations in deposited Pt thickness and the degree of interparticle contact during sintering lead to substantial changes in the conductive pathway density and cross-sectional connectivity. As a result, the measured conductivity reflects composite-level percolative transport rather than intrinsic Pt film quality. The observed range is therefore consistent with expected variations in metallic volume fraction and network connectivity in such systems.

At the same time, we acknowledge that the term "effective electrical conductivity (σ^{eff})" may introduce ambiguity when comparing architectures with fundamentally different geometric definitions. To avoid potential misunderstanding, we have removed all references to "effective electrical conductivity (σ^{eff})" from both the main manuscript and the Supplementary Information. We have also eliminated the direct one-to-one comparison between specific 2D and 3D sample sets that relied on geometry-dependent normalization.

Following the Reviewer 3's suggestion in Comment 3-3 that "A simple calculation based on scaling arguments may strengthen the claim that these materials may overtake either 2D SSE implementations or conventional thermoelectrics," we have reformulated the performance evaluation in terms of device-level maximum output power (P_{max}). Specifically, we now compare the thickness-dependent scaling P_{max} (=$V_{oc}^2/(4R_{int})$), calculated from the open-circuit voltage (V_{oc}) and internal resistance (R_{int}), under explicitly defined and identical lateral dimensions and applied heat-flux conditions, with thickness as the only varying parameter.

In conventional 2D SSE devices, spin-current generation and detection are interfacial processes. The open-circuit voltage follows a thickness dependence of the form $V_{oc} \propto (1 - \exp\left(-\frac{t}{\lambda_m}\right))$, where t is the magnetic layer thickness and λ_m is the magnon diffusion length [*Phys. Rev. Lett.* **115**, 096602 (2015)]. Since R_{int} remains approximately constant with increasing FM thickness, the resulting P_{max} follows

$$P_{max}^{2D} = \frac{V_{oc}^2}{4R_{int}} \propto (1 - \exp(-t/\lambda_m))^2,$$

leading to saturation once $t > \lambda_m$.

In contrast, in the present three-dimensional composite, spin-to-charge conversion occurs throughout the bulk volume via a percolated metallic network. Under fixed lateral dimensions, increasing thickness increases the conductive cross-section, resulting in $R_{int} \propto 1/t$, while the generated electromotive force

($\propto V_{oc}$) does not exhibit interfacial saturation. Consequently, within the experimentally relevant thickness range ($\gg \lambda_m$),

$$P_{max}^{3D} = \frac{V_{oc}^2}{4R_{int}} \propto t,$$

reflecting non-saturating, volumetric scaling behavior.

Importantly, this scaling behavior arises from the volumetric conversion topology of the composite, rather than from a normalization artifact. In conventional interfacial 2D SSE geometries, spin-to-charge conversion is confined to a thin interfacial region, leading to intrinsic thickness saturation governed by the magnon diffusion length. Such saturation is absent in the present volumetric configuration.

This revised scaling analysis is presented in **Fig. 5** of the main text, and the detailed derivation, assumptions, and material parameters are provided in Supplementary Note 5 and Supplementary Table 1. By directly comparing output power rather than conductivity, this framework clarifies the architectural distinction between diffusion-limited interfacial SSE systems and volumetric bulk composites.

To reflect these revisions, the manuscript has been modified as follows:

- We have replaced the term "effective electrical conductivity (σ^{eff})" throughout the main text and Supplementary Information and eliminated direct σ^{eff}-based comparisons between 2D and 3D systems.

- In the Abstract (page 1), the emphasis has been shifted from conductivity comparison to scaling behavior. The revised sentence now reads:

  "Power analysis indicates that the three-dimensional architecture enables scalable volumetric thermoelectric power generation beyond diffusion-limited thin-film SSE geometries."

- The discussion on the validation of SSE signals and exclusion of parasitic effects has been relocated from the Discussion section to the Results section (page 10, line 18 – page 12, line 13) and organized under a new subsection titled "Validation of trans-scale SSE and exclusion of parasitic effects" for improved clarity.

- On page 21, a new Fig. 5 has been added to present the thickness-dependent power scaling analysis.

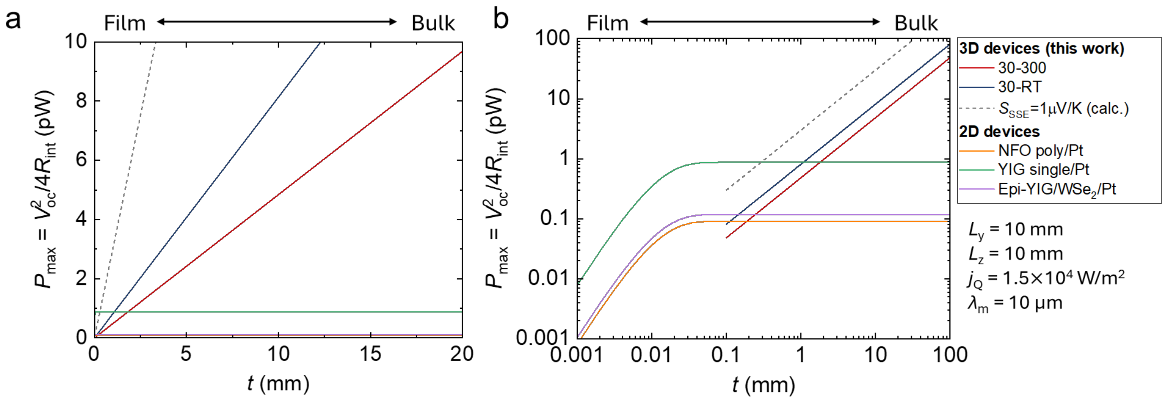

"

Fig. 5| Thickness-dependent scaling of maximum output power in transverse spin Seebeck devices. (a) Linear-scale plot and (b) logarithmic-scale plot of the maximum output power P_{max} as a function of ferromagnetic layer thickness. P_{max}

was calculated under identical device area (L_y = L_z = 10 mm) and heat-flux (j_Q = 1.5×10^4 W/m^2) conditions using $P_{\max} = V_{\rm oc}^2/4R_{\rm int}$, where V_{oc} is the open circuit voltage and R_{int} is the internal resistance. A representative magnon diffusion length of λ_m = 10 μm was used for the 2D reference calculations. The 2D reference systems were modeled using representative material parameters from polycrystalline $NiFe_2O_4$ (NFO poly)/Pt[21], single-crystalline YIG (YIG single)/Pt[68], and epitaxial YIG films (Epi-YIG)/WSe_2/Pt[34] heterostructures reported in the literature. For the 3D architecture, the thickness-dependent scaling is shown only for thicknesses exceeding both λ_m and the experimentally accessible range (> 0.1 mm), thereby representing the volumetric scaling regime. Detailed assumptions and parameter values are provided in Supplementary Note S5."

- On page 12, line 14 – page 14, line 11, a new subsection titled "Thickness-dependent power scaling enabled by volumetric spin conversion" has been introduced to provide a structured discussion of the scaling framework.

"**Thickness-dependent power scaling enabled by volumetric spin conversion**

We evaluate the device-level performance using the maximum output power,

$$P_{\max} = \frac{V_{\rm oc}^2}{4R_{\rm int}}, \tag{4}$$

where V_{oc} is the open-circuit voltage and R_{int} is the internal resistance of the device. To isolate the architectural contribution, we fix the lateral device dimensions (L_y and L_z) and the applied heat-flux boundary condition, while varying only the device or FM thickness t (**Fig. 5**).

For conventional quasi-two-dimensional interfacial SSE devices (**Fig. 1a**), the open-circuit voltage follows $V_{\rm oc}^{\rm 2D}(t) \propto S_0(1-e^{-t/\lambda_m})$. Under fixed NM geometry, the internal resistance remains approximately thickness independent. Therefore,

$$P_{\max}^{\rm 2D}(t) \propto (1-e^{-t/\lambda_m})^2, \tag{5}$$

which saturates for $t \gg \lambda_{\rm m}$. In the present analysis a representative λ_m = 10 μm, as reported for YIG at room temperature[17,67], was adopted for the 2D reference calculations.

In contrast, for the present quasi-three-dimensional composite, the percolated metallic NM network increases the effective conductive cross-section with thickness, resulting in $R_{\rm int}^{\rm 3D} \propto 1/t$. Within the experimentally accessible thickness range ($\gg \lambda_{\rm m}$), the effective SSE is not limited by interfacial magnon diffusion and therefore does not exhibit the saturation characteristic of two-dimensional systems. Consequently,

$$P_{\max}^{\rm 3D}(t) \propto t, \tag{6}$$

leading to a non-saturating increase of output power within increasing thickness. A detailed derivation of these scaling relations, including expressions in terms of experimentally measurable parameters, is provided in **Supplementary Note S5**.

For the three-dimensional composite, experimentally measured transport parameters from the 30-300 and 30-RT samples were used for the scaling analysis, including κ = 1.87 W/mK, σ = 2.31 × 10^3 S/m, and S_{SSE} = 14.8 nV/K for the 30-RT sample. For the quasi-two-dimensional reference systems, representative material parameters reported for polycrystalline $NiFe_2O_4$/Pt[21], single-crystalline YIG/Pt[68], and epitaxial YIG films /WSe_2/Pt[34] heterostructures were adopted, using literature values of S_{SSE}, κ of the FM layer and σ of the NM layer. Using these parameters (see **Table S1**), **Fig. 5** presents P_{max} as a function of thickness under identical device area (10 mm × 10 mm) and heat-flux (j_Q = 1.5 × 10^4 W/m^2) boundary conditions. For the three-dimensional composite, the scaling analysis is shown only in the regime where the thickness exceeds both λ_m and the experimentally accessible range (> 0.1 mm),

corresponding to the volumetric scaling regime. The quasi-three-dimensional composite architecture exhibits an approximately linear increase in output power with thickness, whereas conventional quasi-two-dimensional devices exhibit saturation behavior governed by λ_m. This contrast demonstrates that the present architecture removes the intrinsic interfacial thickness limitation characteristic of thin-film SSE systems and establishes a bulk-operating scaling regime enabled by volumetric spin-to-charge conversion. The dashed lines in **Fig. 5** represent projected scaling behavior assuming higher intrinsic S_{SSE} comparable to those reported in optimized thin-film systems (on the order of 1 μV/K), while maintaining the same three-dimensional architecture and boundary conditions. Because the maximum output power in the volumetric regime follows $P_{max}^{3D} \propto S_{SSE}^2 t$, increasing the intrinsic S_{SSE} enhances the slope of the linear thickness scaling without altering the linear thickness dependence."

- The previous Supplementary Note 4, which defined and discussed the "effective electrical conductivity," has been removed in its entirety. The subsequent Supplementary Notes have been renumbered accordingly.
- On pages 7–8, a new Supplementary Note 5 has been added to present the revised scaling framework and related derivations.

"Note 5. Thickness-dependent scaling of output power in 2D and 3D spin Seebeck devices

General expression for output power

The transverse electric field generated by the SSE is expressed as

$$E_y = S_{SSE} \nabla T,$$

where S_{SSE} is the spin Seebeck coefficient and ∇T is the applied temperature gradient. The open-circuit voltage measured along the device length L_y is therefore

$$V_{oc} = S_{SSE} \nabla T L_y.$$

When the device is connected to an external load resistance R_L, the output power is

$$P(R_L) = \frac{V_{oc}^2 R_L}{(R_{int} + R_L)^2},$$

where R_{int} is the internal resistance of the device. Maximum power transfer occurs when $R_L = R_{int}$, yielding

$$P_{max} = \frac{V_{oc}^2}{4R_{int}}.$$

This expression forms the basis for the thickness-dependent scaling analysis presented below.

Thickness dependence in conventional quasi-two-dimensional SSE systems

In conventional quasi-two-dimensional SSE devices, spin-current generation and spin-to-charge conversion are interfacial processes. The S_{SSE} therefore depends on the magnetic layer thickness t as

$$S_{SSE}(t) = S_0(1 - e^{-t/\lambda_m}),$$

where S_0 is the bulk spin Seebeck coefficient and is λ_m the magnon diffusion length.

The open-circuit voltage becomes

$$V_{oc}^{2D}(t) = S_0(1 - e^{-t/\lambda_m}) \nabla T L_y.$$

Under fixed normal-metal geometry, the internal resistance is approximately independent of the magnetic layer thickness ($R_{int}^{2D} \approx$ const.).

Accordingly, the maximum output power follows

$$P_{max}^{2D}(t) = \frac{\left[S_0\left(1 - e^{-\frac{t}{\lambda_m}}\right) \nabla T L_y\right]^2}{4R_{int}^{2D}} \propto (1 - e^{-t/\lambda_m})^2.$$

As t exceeds λ_m, both the voltage and the output power approach saturation. In the present scaling analysis, we adopt a representative magnon diffusion length of λ_m ~ 10 μm at room temperature as

reported in the literature. We note that additional magnon energy-relaxation mechanisms, which have been discussed at shorter length scales on the order of ~250 nm, are not included in this simplified model. Since the thickness range considered in this work significantly exceeds the sub-micron regime, the diffusion-limited description provides an appropriate approximation for the purpose of architectural scaling comparison.

Thickness dependence in quasi-three-dimensional volumetric SSE composites

In the present three-dimensional composite architecture, spin-to-charge conversion occurs throughout the bulk volume via a percolated metallic network.

Under fixed lateral dimensions L_x and L_y, increasing thickness t increases the effective conductive cross-section of the interconnected metallic pathways. Using σ, the internal resistance scales as

$$R_{\mathrm{int}}^{\mathrm{3D}} \approx \frac{1}{\sigma}\frac{L_y}{L_x t} \propto \frac{1}{t}.$$

Within the experimentally relevant thickness range (> 0.1 mm), the S_{SSE} does not exhibit the interfacial saturation characteristic of quasi-two-dimensional systems. The open-circuit voltage can therefore be expressed as

$$V_{\mathrm{oc}}^{\mathrm{3D}} = S_{\mathrm{SSE}}^{\mathrm{3D}} \nabla T L_y,$$

where $S_{\mathrm{SSE}}^{\mathrm{3D}}$ is approximately thickness-independent in the absence of interfacial limitation.

Substituting into the general power expression yields

$$P_{\max}^{\mathrm{3D}}(t) = \frac{[S_{\mathrm{SSE}}^{\mathrm{3D}} \nabla T L_y]^2}{4R_{\mathrm{int}}^{\mathrm{3D}}} \propto t.$$

Thus, in contrast to the saturation behavior of 2D interfacial SSE systems, the three-dimensional composite architecture exhibits a non-saturating power scaling with thickness within the accessible regime.

Architectural origin of the scaling difference

The key distinction between the two architectures arises from the spatial distribution of spin-to-charge conversion:

- In two-dimensional systems, conversion is confined to a thin interfacial region, leading to intrinsic thickness saturation governed by the magnon diffusion length.
- In the three-dimensional composite, conversion occurs throughout the bulk volume, while the internal resistance decreases with increasing conductive cross-section.

This difference in conversion topology results in fundamentally different thickness-scaling behaviors of output power."

- On pages 13 in Supplementary Information, Supplementary Table 1 has been modified:

"Supplementary Table 1. Material geometries and properties used for the maximum output power calculation. The materials include polycrystalline $NiFe_2O_4$ (NFO), single-crystalline YIG, YIG film grown by liquid-phase epitaxy (Epi-YIG). Blanks (-) indicate properties that are not applicable.

Ref.	Material			Thickness			σ of metallic layers (S/m)	κ (W/mK)	S_{SSE} (nV/K)
	Substrate	FM	NM	Substrate (mm)	FM (mm)	NM (nm)			
[4]	-	NFO polycrystal	Pt	-	0.5	10	1.60×10^6	10.37	330

5	-	YIG single crystal	Pt	-	1	10	5.30×10^6	7.9	428
6	GGG	Epi-YIG	WSe_2/Pt	0.5	0.005	5.7	1.46×10^6	7.8	392
This work	-	YIG polycrystal	Pt	-	~ 1 mm		4.96×10^3 (30-300)	1.80	7.5
							2.31×10^3 (30-RT)	1.87	14.8

”

We believe that these revisions clarify the points raised and strengthen the manuscript accordingly.

[Reply to the comments of Reviewer #3]

(Comment 3)

The manuscript demonstrates the use of a unique powder sputter coating and sintering system that allows for nanoscale metallic networks to formed in a 3D FMI/NM composite material. This turns thermoelectric power conversion from the spin Seebeck effect from a 2D process to a 3D process, which is fundamentally more compatible with applications. I believe this work represents a significant technical feat by the authors, and is an genuine advance to the field. However, I do have some concerns as outlined below:

(Response 3)

We sincerely thank the Reviewer for the thoughtful and encouraging evaluation of our work. We appreciate the recognition of the architectural advance enabled by the powder sputter coating and sintering approach, and the acknowledgment that the transition from a planar to a volumetric spin Seebeck geometry represents a meaningful development for practical implementation.

We also value the constructive concerns raised by the Reviewer. These comments have been instrumental in further clarifying the scope, quantitative interpretation, and physical implications of our study. In response, we have substantially strengthened the manuscript by reformulating the performance comparison using directly measurable quantities, incorporating a scaling analysis to contrast planar and volumetric SSE architectures under equivalent geometric assumptions, and clarifying the treatment of thermal transport and signal origin.

Detailed point-by-point responses to each concern are provided below.

(Comment 3-1)

It is not clear to me that the performance of the 3D composite is significantly better than either conventional thermoelectrics, or 2D SSE thermoelectric systems. Authors point to the enhanced electrical conductivity compared to 2D SSE implementations, but this is only when an "effective" conductivity is used. This effective conductivity is misleading, since in the 2D implementation it counts the mm-scale dimensions of an insulator in the conductivity calculations. Perhaps a much more convincing metric would be a comparison of actual power generation given similar dimensions and thermal gradients, between 2D SSE, 3D SSE, and conventional thermoelectrics.

(Response 3-1)

We thank the Reviewer for this important and constructive comment. We agree that the use of an "effective" electrical conductivity (σ^{eff}) may introduce ambiguity when comparing architectures with fundamentally different geometric definitions. To avoid potential misunderstanding, we have removed all references to "effective electrical conductivity (σ^{eff})" from both the main manuscript and the Supplementary Information. We have also eliminated the direct one-to-one comparison between specific 2D and 3D sample sets that relied on geometry-dependent normalization.

Following the Reviewer's suggestion in Comment 3-3 that "A simple calculation based on scaling arguments may strengthen the claim that these materials may overtake either 2D SSE implementations or conventional thermoelectrics," we have reformulated the performance evaluation in terms of device-level maximum output power (P_{max}). Specifically, we now compare the thickness-dependent scaling P_{max} ($=V_{oc}^2/(4R_{int})$), calculated from the open-circuit voltage (V_{oc}) and internal resistance (R_{int}), under explicitly defined and identical lateral dimensions and applied heat-flux conditions, with thickness as the only varying parameter.

In conventional 2D SSE devices, spin-current generation and detection are interfacial processes. The open-circuit voltage follows a thickness dependence of the form $V_{oc} \propto (1 - \exp\left(-\frac{t}{\lambda_m}\right))$, where t is the magnetic layer thickness and λ_m is the magnon diffusion length [*Phys. Rev. Lett.* **115**, 096602 (2015)]. Since R_{int} remains approximately constant with increasing FM thickness, the resulting P_{max} follows

$$P_{max}^{2D} = \frac{V_{oc}^2}{4R_{int}} \propto (1 - \exp(-t/\lambda_m))^2,$$

leading to saturation once $t > \lambda_m$.

In contrast, in the present three-dimensional composite, spin-to-charge conversion occurs throughout the bulk volume via a percolated metallic network. Under fixed lateral dimensions, increasing thickness increases the conductive cross-section, resulting in $R_{int} \propto 1/t$, while the generated electromotive force ($\propto V_{oc}$) does not exhibit interfacial saturation. Consequently, within the experimentally relevant thickness range ($\gg \lambda_m$),

$$P_{max}^{3D} = \frac{V_{oc}^2}{4R_{int}} \propto t,$$

reflecting non-saturating, volumetric scaling behavior.

Importantly, this scaling behavior arises from the volumetric conversion topology of the composite, rather than from a normalization artifact. In conventional interfacial 2D SSE geometries, spin-to-charge conversion is confined to a thin interfacial region, leading to intrinsic thickness saturation governed by the magnon diffusion length. Such saturation is absent in the present volumetric configuration.

This revised scaling analysis is presented in **Fig. 5** of the main text, and the detailed derivation, assumptions, and material parameters are provided in Supplementary Note 5 and Supplementary Table 1. By directly comparing output power rather than conductivity, this framework clarifies the architectural distinction between diffusion-limited interfacial SSE systems and volumetric bulk composites.

To reflect these revisions, the manuscript has been modified as follows:

- We have replaced the term "effective electrical conductivity (σ^{eff})" throughout the main text and Supplementary Information and eliminated direct σ^{eff}-based comparisons between 2D and 3D systems.
- In the Abstract (page 1), the emphasis has been shifted from conductivity comparison to scaling behavior. The revised sentence now reads:

  "Power analysis indicates that the three-dimensional architecture enables scalable volumetric thermoelectric power generation beyond diffusion-limited thin-film SSE geometries."
- The discussion on the validation of SSE signals and exclusion of parasitic effects has been relocated from the Discussion section to the Results section (page 10, line 18 – page 12, line 13) and organized under a new subsection titled "Validation of trans-scale SSE and exclusion of parasitic effects" for improved clarity.
- On page 21, a new Fig. 5 has been added to present the thickness-dependent power scaling analysis.

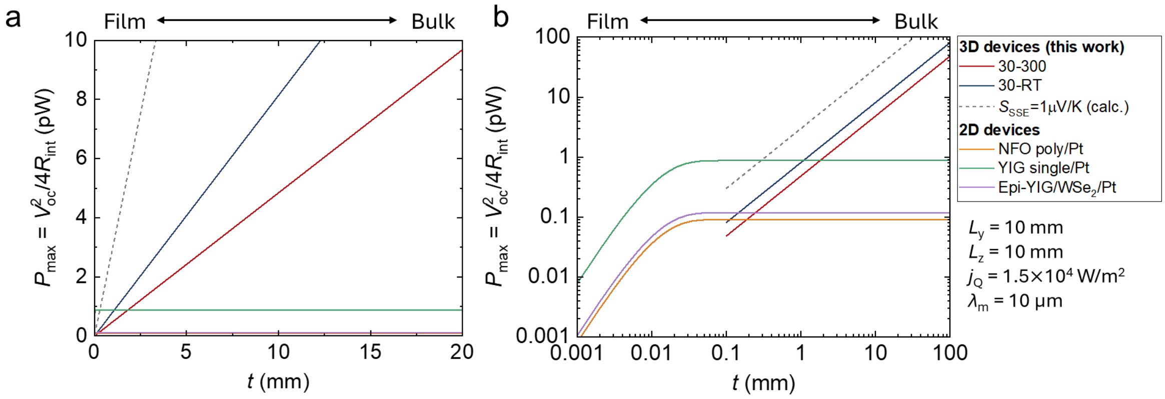

"

Fig. 5| Thickness-dependent scaling of maximum output power in transverse spin Seebeck devices. (a) Linear-scale plot and (b) logarithmic-scale plot of the maximum output power P_{max} as a function of ferromagnetic layer thickness. P_{max} was calculated under identical device area ($L_y = L_z = 10$ mm) and heat-flux ($j_Q = 1.5\times10^4$ W/m^2) conditions using $P_{\text{max}} = V_{\text{oc}}^2/4R_{\text{int}}$, where V_{oc} is the open circuit voltage and R_{int} is the internal resistance. A representative magnon diffusion length of λ_m = 10 μm was used for the 2D reference calculations. The 2D reference systems were modeled using representative material parameters from polycrystalline $NiFe_2O_4$ (NFO poly)/Pt[21], single-crystalline YIG (YIG single)/Pt[68], and epitaxial YIG films (Epi-YIG)/WSe_2/Pt[34] heterostructures reported in the literature. For the 3D architecture, the thickness-dependent scaling is shown only for thicknesses exceeding both λ_m and the experimentally accessible range (> 0.1 mm), thereby representing the volumetric scaling regime. Detailed assumptions and parameter values are provided in Supplementary Note S5."

- On page 12, line 14 – page 14, line 11, a new subsection titled "Thickness-dependent power scaling enabled by volumetric spin conversion" has been introduced to provide a structured discussion of the scaling framework.

"Thickness-dependent power scaling enabled by volumetric spin conversion

We evaluate the device-level performance using the maximum output power,

$$P_{\text{max}} = \frac{V_{\text{oc}}^2}{4R_{\text{int}}}, \quad (4)$$

where V_{oc} is the open-circuit voltage and R_{int} is the internal resistance of the device. To isolate the

architectural contribution, we fix the lateral device dimensions (L_y and L_z) and the applied heat-flux boundary condition, while varying only the device or FM thickness t (**Fig. 5**).

For conventional quasi-two-dimensional interfacial SSE devices (**Fig. 1a**), the open-circuit voltage follows $V_{\mathrm{oc}}^{\mathrm{2D}}(t) \propto S_0(1 - e^{-t/\lambda_m})$. Under fixed NM geometry, the internal resistance remains approximately thickness independent. Therefore,

$$P_{\mathrm{max}}^{\mathrm{2D}}(t) \propto (1 - e^{-t/\lambda_m})^2, \tag{5}$$

which saturates for $t \gg \lambda_{\mathrm{m}}$. In the present analysis a representative λ_{m} = 10 μm, as reported for YIG at room temperature[17,67], was adopted for the 2D reference calculations.

In contrast, for the present quasi-three-dimensional composite, the percolated metallic NM network increases the effective conductive cross-section with thickness, resulting in $R_{\mathrm{int}}^{\mathrm{3D}} \propto 1/t$. Within the experimentally accessible thickness range ($\gg \lambda_{\mathrm{m}}$), the effective SSE is not limited by interfacial magnon diffusion and therefore does not exhibit the saturation characteristic of two-dimensional systems. Consequently,

$$P_{\mathrm{max}}^{\mathrm{3D}}(t) \propto t, \tag{6}$$

leading to a non-saturating increase of output power within increasing thickness. A detailed derivation of these scaling relations, including expressions in terms of experimentally measurable parameters, is provided in **Supplementary Note S5**.

For the three-dimensional composite, experimentally measured transport parameters from the 30-300 and 30-RT samples were used for the scaling analysis, including κ = 1.87 W/mK, σ = 2.31 × 10^3 S/m, and S_{SSE} = 14.8 nV/K for the 30-RT sample. For the quasi-two-dimensional reference systems, representative material parameters reported for polycrystalline $NiFe_2O_4$/Pt[21], single-crystalline YIG/Pt[68], and epitaxial YIG films /WSe_2/Pt[34] heterostructures were adopted, using literature values of S_{SSE}, κ of the FM layer and σ of the NM layer. Using these parameters (see **Table S1**), **Fig. 5** presents P_{max} as a function of thickness under identical device area (10 mm × 10 mm) and heat-flux (j_{Q} = 1.5 × 10^4 W/m^2) boundary conditions. For the three-dimensional composite, the scaling analysis is shown only in the regime where the thickness exceeds both λ_{m} and the experimentally accessible range (> 0.1 mm), corresponding to the volumetric scaling regime. The quasi-three-dimensional composite architecture exhibits an approximately linear increase in output power with thickness, whereas conventional quasi-two-dimensional devices exhibit saturation behavior governed by λ_{m}. This contrast demonstrates that the present architecture removes the intrinsic interfacial thickness limitation characteristic of thin-film SSE systems and establishes a bulk-operating scaling regime enabled by volumetric spin-to-charge conversion. The dashed lines in **Fig. 5** represent projected scaling behavior assuming higher intrinsic S_{SSE} comparable to those reported in optimized thin-film systems (on the order of 1 μV/K), while maintaining the same three-dimensional architecture and boundary conditions. Because the maximum output power in the volumetric regime follows $P_{\mathrm{max}}^{\mathrm{3D}} \propto S_{\mathrm{SSE}}^2 t$, increasing the intrinsic S_{SSE} enhances the slope of the linear thickness scaling without altering the linear thickness dependence."

- The previous Supplementary Note 4, which defined and discussed the "effective electrical conductivity," has been removed in its entirety. The subsequent Supplementary Notes have been renumbered accordingly.
- On pages 7–8, a new Supplementary Note 5 has been added to present the revised scaling framework and related derivations.

“Note 5. Thickness-dependent scaling of output power in 2D and 3D spin Seebeck devices

General expression for output power

The transverse electric field generated by the SSE is expressed as

$$E_y = S_{\mathrm{SSE}} \nabla T,$$

where S_{SSE} is the spin Seebeck coefficient and ∇T is the applied temperature gradient. The open-circuit voltage measured along the device length L_y is therefore

$$V_{\mathrm{oc}} = S_{\mathrm{SSE}} \nabla T L_y.$$

When the device is connected to an external load resistance R_L, the output power is

$$P(R_L) = \frac{V_{\mathrm{oc}}^2 R_L}{(R_{\mathrm{int}} + R_L)^2},$$

where R_{int} is the internal resistance of the device. Maximum power transfer occurs when $R_L = R_{\mathrm{int}}$, yielding

$$P_{\max} = \frac{V_{\mathrm{oc}}^2}{4R_{\mathrm{int}}}.$$

This expression forms the basis for the thickness-dependent scaling analysis presented below.

Thickness dependence in conventional quasi-two-dimensional SSE systems

In conventional quasi-two-dimensional SSE devices, spin-current generation and spin-to-charge conversion are interfacial processes. The S_{SSE} therefore depends on the magnetic layer thickness t as

$$S_{\mathrm{SSE}}(t) = S_0(1 - e^{-t/\lambda_m}),$$

where S_0 is the bulk spin Seebeck coefficient and is λ_m the magnon diffusion length.

The open-circuit voltage becomes

$$V_{\mathrm{oc}}^{2\mathrm{D}}(t) = S_0(1 - e^{-t/\lambda_m}) \nabla T L_y.$$

Under fixed normal-metal geometry, the internal resistance is approximately independent of the magnetic layer thickness ($R_{\mathrm{int}}^{2\mathrm{D}} \approx$ const.).

Accordingly, the maximum output power follows

$$P_{\max}^{2\mathrm{D}}(t) = \frac{\left[S_0\left(1 - e^{-\frac{t}{\lambda_m}}\right) \nabla T L_y\right]^2}{4R_{\mathrm{int}}^{2\mathrm{D}}} \propto (1 - e^{-t/\lambda_m})^2.$$

As t exceeds λ_m, both the voltage and the output power approach saturation. In the present scaling analysis, we adopt a representative magnon diffusion length of λ_m ~ 10 μm at room temperature as reported in the literature. We note that additional magnon energy-relaxation mechanisms, which have been discussed at shorter length scales on the order of ~250 nm, are not included in this simplified model. Since the thickness range considered in this work significantly exceeds the sub-micron regime, the diffusion-limited description provides an appropriate approximation for the purpose of architectural scaling comparison.

Thickness dependence in quasi-three-dimensional volumetric SSE composites

In the present three-dimensional composite architecture, spin-to-charge conversion occurs throughout the bulk volume via a percolated metallic network.

Under fixed lateral dimensions L_x and L_y, increasing thickness t increases the effective conductive cross-section of the interconnected metallic pathways. Using σ, the internal resistance scales as

$$R_{\mathrm{int}}^{3\mathrm{D}} \approx \frac{1}{\sigma} \frac{L_y}{L_x t} \propto \frac{1}{t}.$$

Within the experimentally relevant thickness range (> 0.1 mm), the S_{SSE} does not exhibit the interfacial saturation characteristic of quasi-two-dimensional systems. The open-circuit voltage can therefore be expressed as

$$V_{\mathrm{oc}}^{3\mathrm{D}} = S_{\mathrm{SSE}}^{3\mathrm{D}} \nabla T L_y,$$

where $S_{\mathrm{SSE}}^{\mathrm{3D}}$ is approximately thickness-independent in the absence of interfacial limitation. Substituting into the general power expression yields

$$P_{\max}^{\mathrm{3D}}(t) = \frac{[S_{\mathrm{SSE}}^{\mathrm{3D}} \nabla T L_y]^2}{4R_{\mathrm{int}}^{\mathrm{3D}}} \propto t.$$

Thus, in contrast to the saturation behavior of 2D interfacial SSE systems, the three-dimensional composite architecture exhibits a non-saturating power scaling with thickness within the accessible regime.

Architectural origin of the scaling difference

The key distinction between the two architectures arises from the spatial distribution of spin-to-charge conversion:

- In two-dimensional systems, conversion is confined to a thin interfacial region, leading to intrinsic thickness saturation governed by the magnon diffusion length.
- In the three-dimensional composite, conversion occurs throughout the bulk volume, while the internal resistance decreases with increasing conductive cross-section.

This difference in conversion topology results in fundamentally different thickness-scaling behaviors of output power."

- On pages 13 in Supplementary Information, Supplementary Table 1 has been modified:

"Supplementary Table 1. Material geometries and properties used for the maximum output power calculation. The materials include polycrystalline $NiFe_2O_4$ (NFO), single-crystalline YIG, YIG film grown by liquid-phase epitaxy (Epi-YIG). Blanks (-) indicate properties that are not applicable.

Ref.	Material			Thickness			σ of metallic layers (S/m)	κ (W/mK)	S_{SSE} (nV/K)
	Substrate	FM	NM	Substrate (mm)	FM (mm)	NM (nm)			
[4]	-	NFO polycrystal	Pt	-	0.5	10	1.60×10^6	10.37	330
[5]	-	YIG single crystal	Pt	-	1	10	5.30×10^6	7.9	428
[6]	GGG	Epi-YIG	WSe_2/Pt	0.5	0.005	5.7	1.46×10^6	7.8	392
This work	-	YIG polycrystal	Pt	-	~ 1 mm		4.96×10^3 (30-300)	1.80	7.5
							2.31×10^3 (30-RT)	1.87	14.8

"

We thank the Reviewer for this valuable suggestion. The geometry-controlled power scaling analysis provides a transparent comparison based on output power and clarifies the fundamental distinction

between interfacial and volumetric spin thermoelectric architectures. We believe that this revision has strengthened the manuscript by more clearly highlighting the architectural origin of the performance difference.

(Comment 3-2)

I am also concerned with the treatment of the 3D composite material as a thermally isotropic system, whereas the metallic nanostructure makes it highly anisotropic in thermal conductivity. Due to the thermal conductivity mismatch between the metallic layers and the insulating layers, would it not be expected that some of the thermal gradient be dropped across the metallic regions and therefore generate no SSE? For example, if a magnetic thin film was used to coat a powdered non-magnetic insulator, would we expect to see a large ANE signal still? Would a combination of both effects lead to compounding gains?

(Response 3-2)

We sincerely thank the reviewer for raising this important point. We address the concerns below.

(Comment 3-2a – thermal isotropy of the composite)

I am also concerned with the treatment of the 3D composite material as a thermally isotropic system, whereas the metallic nanostructure makes it highly anisotropic in thermal conductivity.

(Response 3-2a)

We thank the Reviewer for raising this important point regarding possible thermal anisotropy.

The composite is fabricated by sintering randomly distributed Pt-coated YIG powders at the millimeter scale. As a result, the local FMI/NM interfaces are statistically oriented without long-range alignment. While local anisotropy may exist at the level of individual coated particles, such anisotropy is statistically averaged out at the bulk scale and therefore becomes negligible due to the random orientation distribution. Accordingly, the composite can be regarded as effectively isotropic for device-level thermoelectric analysis. Although Pt possesses higher intrinsic thermal conductivity than YIG, its nanoscale thickness and random spatial distribution within the composite prevent the emergence of macroscopic thermal anisotropy.

The measured spin Seebeck coefficient under different magneto-thermal configurations further supports this interpretation. Specifically, the transverse SSE signals measured along two orthogonal directions (**Figs. 3b** and **3d**) exhibit comparable magnitudes (14.8 nV/K for H_z-$\nabla_x T$ configuration and 14.4 nV/K for H_x-$\nabla_z T$ configuration). This directional invariance indicates that any residual anisotropy in thermal conductivity or spin transport does not measurably influence the observed macroscopic SSE response.

For clarify, we have modified the manuscript as follows:

- On page 23, lines 13–15, we added the following sentence:

  "Owing to the statistically random orientation of Pt-coated YIG particles in the sintered composite, the macroscopic thermal and spin transport properties are treated as effectively isotropic in the device-level analysis."

(Comment 3-2b – thermal conductivity mismatch and temperature-gradient redistribution)

Due to the thermal conductivity mismatch between the metallic layers and the insulating layers, would it not be expected that some of the thermal gradient be dropped across the metallic regions and therefore generate no SSE?

(Response 3-2b)

We thank the Reviewer for raising this point regarding the possible redistribution of the temperature gradient due to thermal conductivity mismatch between the metallic and insulating regions.

Although metallic Pt possesses a higher intrinsic thermal conductivity than oxide YIG, its thickness in the present composite is limited to 15–30 nm as a coating layer on each particle. Under cross-plane heat-flow conditions, the thermal resistance of a layer scales as $R_{th} = t/(\kappa A)$, where t is the thickness, κ is the thermal conductivity and A is the cross-sectional area. Owing to the nanoscale thickness of the Pt coating, its cross-plane thermal resistance remains much smaller than that of the thicker YIG region despite its higher intrinsic κ.

As a result, under an applied heat boundary condition, the dominant portion of the temperature drop occurs across the YIG phase rather than being short-circuited through the metallic coating. Therefore, the effective temperature gradient driving the SSE is primarily established within the magnetic insulator.

In addition, several experimental observations confirm the SSE origin of the measured signal. The transverse voltage is suppressed at high magnetic fields consistent with magnon-related behavior (**Fig. 3f**), and no anomalous Hall contribution is detected from the Pt network (**Fig. 3e**). Furthermore, replacing Pt with W leads to a reversal of the voltage sign consistent with the opposite spin Hall angle of W (**Fig. 4**), providing additional evidence that the measured voltage arises from spin-to-charge conversion rather than parasitic thermoelectric effects in the metallic regions.

Taken together, although local thermal conductivity mismatch exists at the particle level, it does not lead to a significant suppression of the effective temperature gradient within the magnetic phase, nor does it alter the SSE-dominated macroscopic response of the composite.

For clarity and to better address the Reviewer's concern, the discussion on the validation of SSE origin and exclusion of parasitic thermoelectric contributions has been relocated from the Discussion section to the Results section (currently on page 10, line 17 – page 12, line 12) and organized as an independent subsection "Validation of trans-scale SSE and exclusion of parasitic effects."

(Comment 3-2c – case of magnetic conductive coating)

If a magnetic thin film was used to coat a powdered non-magnetic insulator, would we expect to see a large ANE signal still? Would a combination of both effects lead to compounding gains?

(Response 3-2c)

We thank the Reviewer for these insightful questions regarding the hypothetical case of a magnetic conductive coating and the possible coexistence of multiple transverse thermoelectric contributions.

If the coating layer were both ferromagnetic and electrically conductive, two distinct transverse voltage contributions could, in principle, arise under an applied temperature gradient.

First, the ANE would generate a transverse electric field within the magnetic conductive coating itself. Second, a thermal spin current, originating from the ferromagnetic insulator (FMI) via the SSE, could

be converted into a transverse charge current within the ferromagnetic metal (FMM) coating through the spin-to-charge conversion, including anomalous ISHE. In this case, spin-to-charge conversion would occur directly within the FMM coating without requiring a separate NM layer.

As the Reviewer correctly noted, both the ANE-induced electric field and the SSE-driven spin-to-charge conversion are governed by the same transverse device geometry. The total measured transverse electric field in a complex nanostructured composite corresponds to the superposition of all allowed symmetry-permitted contributions, as formulated in **Equations (1)–(3)** of the Introduction and **Supplementary Note 1**. Therefore, in principle, ANE- and SSE-related contributions may add constructively and yield enhanced output when their signs and geometrical factors are aligned.

Indeed, hybrid thermoelectric conversion combining SSE and ANE has been reported in YIG/FMM bilayer systems [see Fig. 12 in Ref. 9: *Proc. IEEE*, **104** 1946-1973 (2016)], where a FMM such as Ni simultaneously exhibits anomalous Nernst response and spin-to-charge conversion capabilities. These studies demonstrate that coexistence and constructive interplay between ANE and SSE can be engineered in appropriately designed heterostructures.

In the present work, the coating layer is Pt, which is a non-ferromagnetic NM. Our measurements show no anomalous Hall term in the transverse resistance (**Fig. 3e**), and the voltage polarity reverses upon replacing Pt with W, consistent with the opposite spin Hall angle of W. These observations indicate that the measured transverse voltage originates from SSE-driven spin-to-charge conversion rather than from an ANE contribution of the metallic network.

To clarify this design perspective, we have explicitly expanded the Discussion section to highlight the potential coexistence and engineering of multiple transverse thermoelectric mechanisms in future composite architectures.

- On page 15, lines 14–23, we added:

"Moreover, hybridization with other transverse thermoelectric phenomena, such as the ANE[7,10,43,80], which has recently shown remarkable performance enhancements in topological materials[81–85] and structurally heterogeneous systems[45,86–88], may provide an additional route for enhancing bulk-scale performance. Hybrid transverse thermoelectric responses combining SSE and ANE have been reported in YIG/FMM bilayer systems, for example using Ni as the ferromagnetic metal[9], where anomalous Nernst response and spin-to-charge conversion coexist within the same device geometry. In this context, employing FMMs that exhibit both a sizable ANE and efficient spin-to-charge conversion, including anomalous inverse spin Hall effect[89], could enable constructive coexistence of ANE- and SSE-driven transverse voltages within the same device geometry. Because both effects share identical symmetry with respect to magnetization and thermal gradient, their contributions may, in principle, superpose and yield synergistic enhancement when their signs and geometrical factors are aligned."

(Comment 3-3)

Some discussion is needed to compare the different benefits a 3D spin thermoelectric material may have over conventional 2D SSE geometries. Even if authors do not scale the devices for production, it would be useful to understand what new geometries and form factors are available that were not available before. A simple calculation based on scaling arguments may strengthen the claim that these materials may overtake either 2D SSE implementations or conventional thermoelectrics.

(Response 3-3)

We thank the Reviewer for this constructive suggestion. As the Reviewer pointed out, a scaling-based comparison provides a clearer framework for assessing the potential advantages of three-dimensional spin thermoelectric architectures over conventional interfacial thin-film SSE geometries. Accordingly, we have added a new thickness-dependent power scaling analysis in **Fig. 5**, and the detailed assumptions and parameters are provided in **Supplementary Note 5** and **Supplementary Table 1** (see also **Response 3-1** for the full derivation and discussion).

Beyond the scaling analysis, the bulk composite architecture removes the interfacial thickness limitation inherent to diffusion-limited thin-film SSE systems. Because spin-to-charge conversion occurs throughout the three-dimensional volume, the output power increases with device thickness without saturation. This establishes a volume-defined architecture in which performance is governed by device thickness rather than by nanoscale diffusion lengths.

To explicitly describe this architectural novelty, we have revised the manuscript as follows:

- On page 16, lines 15–18, we added:

"Unlike interfacial thin-film SSE systems, the present architecture supports bulk-scalable geometries in which output power increases with device thickness without interfacial saturation, thereby expanding the accessible design space for transverse thermoelectric energy conversion."

[Reply to the editor's and reviewers' comments]

We sincerely thank the handling editor and the reviewers for taking the time to review our manuscript and for providing thoughtful and constructive feedback. Below, we provide point-by-point responses to each of the reviewers' comments, along with the corresponding modifications made to the manuscript.

[Reply to the comments of Reviewer #2]

(Comment 2-1)

From Fig. 3, the Ey of YIG-Pt bulk composite in 10 mm size is about 100 μV/m under 5.8 K/mm, thus the transverse thermovoltage is about 1 μV. From the noise in Fig. 4, the transverse thermovoltage in the YIG-W bulk composite can be estimated as small as about 0.1 μV. The paper shows only two samples, and the revised paper does not include additional better experimental results. I worry about the reproducibility and stability of the spin Seebeck effect in bulk composites.

(Response 2-1)

We respectfully clarify that the YIG-W experiment shown in Fig. 4 was designed to verify the physical origin of the observed transverse voltage through the sign reversal expected from the negative spin Hall angle of W. This experiment was performed in direct response to the Reviewer's comment in the previous review round: "To conclude the spin Seebeck and inverse spin Hall effects, the results should be also shown in the YIG bulk composites with nonmagnetic metal of negative spin Hall angle such as Ta and W." Thus, the purpose of the YIG-W measurement was to confirm the SSE-driven inverse spin Hall origin of the signal, rather than to demonstrate improved device performance or larger signal magnitude.

Despite the smaller signal amplitude in the YIG-W control sample, the polarity reversal and magnetic-field symmetry are clearly resolved. These observations confirm the role of Fig. 4 as a mechanism-validation experiment and support the SSE-driven spin-to-charge conversion origin of the measured transverse voltage.

Regarding reproducibility and stability, we respectfully note that the present manuscript already includes systematic structural, magnetic, and transverse thermoelectric characterization for four independent YIG-Pt bulk composites: 15-300, 15-RT, 30-300, and 30-RT. In particular, for the representative 30-RT sample, Fig. 3 presents measurements obtained under reversed temperature-gradient and magnetization directions, all of which consistently reproduce the expected symmetry and polarity of the SSE signal. These results confirm the reproducibility and stability of the observed bulk SSE response. Thus, the reproducibility and stability of the bulk SSE response are demonstrated primarily by the systematic YIG-Pt composite data, rather than by the YIG-W control experiment.

- To more explicitly state the reproducibility of our data, we have revised the following sentence in the main text (page 10, line 21– page 11, line 1):

  "This consistency supports the assignment of the signal to SSE and demonstrates the reproducibility of the bulk SSE response under orthogonal magnetothermal configurations,

while confirming the directional isotropy of the system and its potential for energy harvesting applications.”

- To more explicitly describe the larger noise level in the YIG-W data, we have revised the sentence in the main text (page 12, lines 13–17):

  “**Figure 4** shows that the transverse thermoelectric voltage of the YIG–W composite in the H_z-$\nabla_x T$ configuration exhibits a clear sign reversal relative to the YIG–Pt composite under otherwise identical conditions, despite the higher noise level associated with the larger electrical resistance of the control sample (**Supplementary Note 4**).”

(Comment 2-2)

The spin Seebeck coefficient of YIG-Pt bulk composite is only 14 nV/K, 30 times smaller than 428 nV/K in conventional YIG/Pt. The bulk composite does not have better performance the conversional spin Seebeck device. What is internal resistance of bulk composite ? The power of bulk composite is as small as pW and could be over-estimated.

(Response 2-2)

We respectfully clarify that the purpose of the present work is not to demonstrate record spin Seebeck coefficients or maximum thermoelectric output from the current material system. Rather, the central advance of this study is the experimental establishment of a bulk-operating volumetric scaling regime for spin thermoelectric conversion, which fundamentally differs from the thickness-saturating behavior of conventional interfacial SSE devices.

Accordingly, direct comparison of the absolute S_{SSE} value of the present bulk composite with thin-film YIG/Pt systems, which have been optimized over a decade, does not appropriately reflect the architectural significance of the present work. As explicitly shown in the newly added Fig. 5, conventional two-dimensional SSE systems exhibit intrinsic thickness saturation governed by magnon diffusion, whereas the present three-dimensional architecture exhibits non-saturating thickness-dependent scaling of the maximum output power P_{max}.

The quantities used to calculate $R_{int}^{3D}(t)$ and $P_{max}^{3D}(t)$ are explicitly provided in the main text, Supplementary Note 5 (Equations S15–S17), and Supplementary Table 1. Specifically, $R_{int}^{3D}(t)$ was calculated from the measured electrical conductivity and defined device geometry as $R_{int}^{3D} \approx \frac{1}{\sigma}\frac{L_y}{L_x t}$ (Equation S15 in the Supplementary Information), and $P_{max}^{3D}(t)$ was obtained using Equation 4 in the main text ($P_{max} = \frac{V_{oc}^2}{4R_{int}}$). Thus, the thickness dependence of the power output is calculated from experimentally measured transport parameters and explicitly defined geometric conditions, rather than from an arbitrary estimate.

As a simple order-of-magnitude check, using the measured parameters of the 30-RT sample gives σ ~ 10^3 S/m, S_{SSE} ~ 10^{-8} V/K, j_Q ~ 10^4 W/m^2, κ ~ 1 W/mK, and $L_x L_y$ ~ 10^{-4} m^2. Substituting these orders into $P_{max} = \sigma S_{SSE}^2 j_Q^2 L_x L_y t/(4\kappa^2)$ gives a sub-pW power scale for t ~ 10^{-3} m, consistent with the scale shown in Fig. 5. This confirms that the calculated power scale is internally consistent with the measured transport parameters and is not overestimated.

We therefore respectfully submit that a comparison based only on absolute material coefficients does not capture the central novelty of this work, which lies in the experimentally demonstrated volumetric scaling architecture for bulk SSE systems.

[Reply to the comments of Reviewer #3]

(Comment 3)

The authors have fully addressed my concerns and I believe the manuscript is ready for publication at this point. I appreciated the additional of the scaling analysis, as it really enhances the clarity of presentation and highlights why this result is as transformative as it is.

(Response 3)

We sincerely thank the Reviewer for the careful evaluation of our revised manuscript and for the positive assessment of our work. We are particularly grateful that the added thickness-dependent power-scaling analysis clarified the architectural significance and volumetric scaling behavior of the present three-dimensional SSE system. The Reviewer's constructive comments have been very helpful in improving the clarity and presentation of the manuscript.

[Reply to the comments of Reviewer #4]

(Comment 4)

This work proposes and demonstrates a trans-scale spin Seebeck effect (SSE) in 3D YIG-Pt bulk composites, overcoming the thickness saturation limitation of conventional 2D thin-film SSE. The fabrication method, orthogonal magnetothermal configuration measurements, Pt/W contrast experiment, and power scaling analysis are well designed. I have read carefully the revised manuscript and the previous Reviewers' reports. I think the authors have better respond all the questions from Reviewers 2 and 3, and made responding revisions in the revised manuscript. I have only additional two questions before recommendation for publication in Nat Commun.

(Response 4)

We sincerely thank the Reviewer for the careful evaluation of our revised manuscript and the previous reviewers' reports. We are grateful for the positive assessment of our work. We also appreciate the Reviewer's recognition that the revised manuscript has addressed the previous concerns raised by Reviewers #2 and #3. Below, we respond to the two additional questions in detail and have revised the manuscript accordingly.

(Comment 4-1)

The low thermal conductivity of the composite is attributed to interfacial phonon scattering, but lacks quantitative/qualitative decomposition from porosity, interface defects and grain boundary scattering. More detailed mechanism analysis is needed.

(Response 4-1)

We thank the Reviewer for this insightful comment. We agree that the origin of the reduced thermal conductivity should be discussed more carefully.

The measured thermal conductivity of the composites is 1.46–1.87 W/mK, corresponding to only 30–38% of the reported value for polycrystalline YIG (~ 5 W/mK), although the relative density of the representative 30-RT sample is approximately 75%. This large reduction cannot be explained solely by the reduced density and suggests additional phonon-scattering processes introduced by the composite structure.

At the same time, a quantitative decomposition into porosity, interface defects, grain boundaries, and YIG/Pt Kapitza (i.e., interfacial) thermal resistance is difficult at the present stage. In insulating YIG, heat conduction is mainly governed by phonons and, to a lesser extent at room temperature in polycrystalline samples, magnons. Within a Matthiessen picture, the relevant phonon-scattering channels include Umklapp scattering, grain-boundary scattering, interface scattering, defect scattering, and porosity-related scattering. Among these, YIG/Pt interfaces, defects generated during coating and densification, and porosity are expected to be strongly modified in the present composite. However, these contributions have weak and partially overlapping temperature dependences, making a unique quantitative separation difficult from the present thermal-conductivity data alone. In addition, porosity-related phonon scattering depends not only on the porosity fraction but also on pore morphology and connectivity, which further complicates a simple decomposition.

We therefore agree that a full quantitative decomposition of the thermal-resistance sources would require dedicated structural characterization and thermal-transport modeling beyond the scope of the present work. To address the Reviewer's comment, we have revised the manuscript to discuss additional possible scattering mechanisms, including porosity, grain boundaries, defects, and YIG/Pt interfacial thermal resistance.

- We have added the following sentence to the main text (page 8, line 18–page 9, line 2):

  "The reduction likely reflects multiple phonon-scattering channels introduced by the composite structure, including porosity, grain boundaries, interface defects, and thermal boundary resistance at the YIG/Pt interfaces. In insulating YIG, heat conduction is mainly governed by phonons, with a relatively small magnon contribution expected at room temperature in polycrystalline samples[46,54]. Therefore, controlling the YIG/Pt interfacial density, defect structure, and pore morphology may provide an additional route for thermal-conductivity engineering in bulk SSE composites."

(Comment 4-2)

Although XRD and magnetization measurements rule out major magnetic impurity phases, the possible magnetic proximity effect and local strain-induced magnetic order at YIG/Pt interface need further discussion.

(Response 4-2)

We thank the Reviewer for this important comment. We agree that possible interfacial magnetic modifications at YIG/Pt interfaces should be discussed more explicitly.

Magnetic proximity effects at Pt/YIG interfaces could, in principle, induce local spin polarization in Pt near the interface. However, if such an induced magnetic metallic region generated a sizable anomalous Nernst contribution, it would also be expected to produce a detectable anomalous Hall effect. In the present composites, no anomalous Hall contribution is observed in the transverse resistance

measurements (Fig. 3e). Therefore, even if proximity-induced spin polarization exists locally at the YIG/Pt interface, its contribution to transverse transport signals, including the anomalous Hall and anomalous Nernst effects, is negligibly small in our system.

Regarding local strain-induced magnetic order, we agree that strain at YIG/Pt interfaces can affect magnetic anisotropy, spin transparency, and spin-to-charge conversion efficiency. Recent studies on strained Pt/YIG bilayers have shown that strain-induced changes in YIG magnetic anisotropy can enhance spin pumping and SSE-related spin-to-charge conversion [*J. Phys. D: Appl. Phys.* **59**, 025302 (2026)]. Although such local interfacial effects may not be directly captured by SQUID-VSM measurements, they may influence the magnitude of the SSE signal in the present nanostructured composites. However, this mechanism is distinct from a parasitic ANE contribution from a ferromagnetic conductive phase. In our case, the absence of anomalous Hall signals, the Pt/W sign reversal, and the symmetry of the thermoelectric response collectively indicate that the dominant signal is magnon-mediated SSE followed by ISHE-driven spin-charge conversion.

To address the Reviewer's comment, we have revised the manuscript to discuss possible magnetic proximity and strain-induced interfacial effects, while clarifying that these effects may modify spin transparency or SSE efficiency but do not change the assignment of the observed voltage to SSE-driven ISHE.

- We have added the following sentence to the main text (page 11, lines 20–23):

  "Consistently, SQUID-VSM measurements show no additional magnetic component or anomalous magnetic behavior. Even if local strain-induced interfacial modification exists below the detection limit, it would affect interfacial spin transparency or SSE-driven ISHE efficiency[65] rather than generate an independent ANE-like voltage."

- The corresponding reference has been added as Ref. 65 [*J. Phys. D: Appl. Phys.* **59**, 025302 (2026)].
